# Re-assessment of monoclonal antibodies against diclofenac for their application in the analysis of environmental waters†

**DOI:** 10.1039/d3ay01333b

**Published:** 2024-05-14

**Authors:** Stephan Schmidt, Holger Hoffmann, Leif-Alexander Garbe, Andrea Harrer, Markus Steiner, Martin Himly, Rudolf J. Schneider

**Affiliations:** a Department of Analytical Chemistry, Reference Materials, BAM Federal Institute for Materials Research and Testing Richard-Willstätter-Str. 11 12489 Berlin Germany rudolf.schneider@bam.de +49 3081041151; b Technische Universität Berlin Straße des 17. Juni 135 D-10623 Berlin Germany; c Department of Chemistry, Humboldt-Universität zu Berlin Brook-Taylor-Str. 2 D-12489 Berlin Germany; d Hochschule Neubrandenburg, Fachbereich Agrarwirtschaft und Lebensmittelwissenschaften D-17033 Neubrandenburg Germany; e Department of Biosciences and Medical Biology, Division of Allergy and Immunology, Paris Lodron University of Salzburg A-5020 Salzburg Austria

## Abstract

The non-steroidal anti-inflammatory drug (NSAID) diclofenac (DCF) is an important environmental contaminant occurring in surface waters all over the world, because, after excretion, it is not adequately removed from wastewater in sewage treatment plants. To be able to monitor this pollutant, highly efficient analytical methods are needed, including immunoassays. In a medical research project, monoclonal antibodies against diclofenac and its metabolites had been produced. Based on this monoclonal anti-DCF antibody, a new indirect competitive enzyme-linked immunosorbent assay (ELISA) was developed and applied for environmental samples. The introduction of a spacer between diclofenac and the carrier protein in the coating conjugate led to higher sensitivity. With a test midpoint of 3 μg L^−1^ and a measurement range of 1–30 μg L^−1^, the system is not sensitive enough for direct analysis of surface water. However, this assay is quite robust against matrix influences and can be used for wastewater. Without adjustment of the calibration, organic solvents up to 5%, natural organic matter (NOM) up to 10 mg L^−1^, humic acids up to 2.5 mg L^−1^, and salt concentrations up to 6 g L^−1^ NaCl and 75 mg L^−1^ CaCl_2_ are tolerated. The antibody is also stable in a pH range from 3 to 12. Cross-reactivity (CR) of 1% or less was determined for the metabolites 4′-hydroxydiclofenac (4′-OH-DCF), 5-hydroxydiclofenac (5-OH-DCF), DCF lactam, and other NSAIDs. Relevant cross-reactivity occurred only with an amide derivative of DCF, 6-aminohexanoic acid (DCF-Ahx), aceclofenac (ACF) and DCF methyl ester (DCF-Me) with 150%, 61% and 44%, respectively. These substances, however, have not been found in samples. Only DCF-acyl glucuronide with a cross-reactivity of 57% is of some relevance. For the first time, photodegradation products were tested for cross-reactivity. With the ELISA based on this antibody, water samples were analysed. In sewage treatment plant effluents, concentrations in the range of 1.9–5.2 μg L^−1^ were determined directly, with recoveries compared to HPLC-MS/MS averaging 136%. Concentrations in lakes ranged from 3 to 4.4 ng L^−1^ and were, after pre-concentration, determined with an average recovery of 100%.

## Abstract 2

A formerly developed antibody against diclofenac was re-assessed for its application in an indirect competitive ELISA to determine the pharmaceutical in wastewater. A series of matrix influences and cross-reactivities have been investigated.

## Graphical abstract

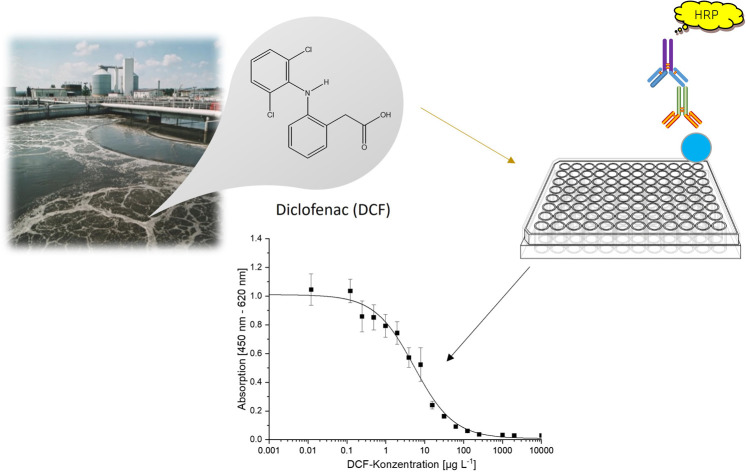

## Introduction

**Fig. 1:**
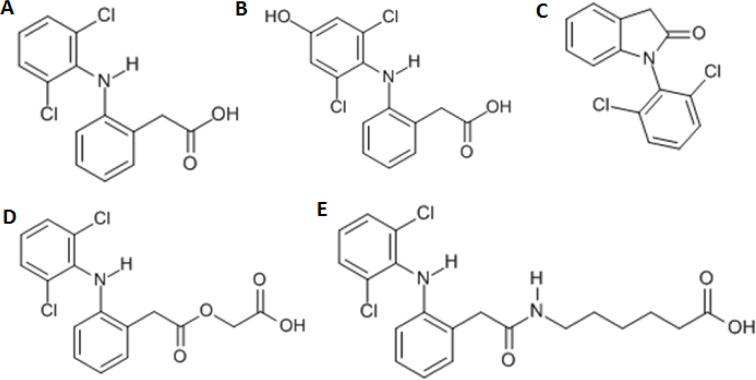
Chemical structures of diclofenac and some derivates: (A) diclofenac, (B) 4′-hydroxydiclofenac, (C) DCF lactam, (D) aceclofenac, (E) DCF-Ahx (Ahx = 6-aminohexanoic acid).

For many years, immunoassays have been used in environmental analysis for the detection and quantification of pharmacologically active compounds and anthropogenic markers. Compared to classical analytical methods, like HPLC or GC, immunoassays are cost-effective, time-saving, and parallelisable. Moreover, they require little sample preparation. With some formats, analysis is possible at the sampling site. Examples of the substances studied are caffeine,^1^ carbamazepine,^2^ cetirizine,^3^ isolithocholic acid,^4^ sulfonamides,^5^ picoxystrobin,^6^ TNT,^7^ flumequine,^8^ and diclofenac^9–11^ (DCF, see Fig. 1A).

DCF is an inflammation inhibitor based on the reversible inhibition of cyclooxygenase.^12,13^ It is used in human medicine as well as in veterinary medicine. DCF is administered in the form of pills or ointments. Orally absorbed DCF is excreted to 65–70% *via* urine and 20–30% *via* faeces. It is largely metabolized. The main metabolite is 4′-hydroxydiclofenac (4′-OH-DCF, see Fig. 1B), which is produced by action of the enzyme CYP2C9.^14–18^

Of ointments, only 6% DCF is absorbed through the skin, the rest is washed off. Owing to the wash-off of ointments, by excretions and improper disposal into toilets, DCF appears in wastewater and finally in sewage treatment plants. However, DCF is only insufficiently removed from wastewaters with an elimination rate of 21–40%.^19^

DCF accumulates along the food chain and is harmful for many species, for example fish,^20–24^ or the almost exterminated Indian vulture.^25,26^ Toxic effects on the liver and kidneys have already been observed in the μg L^−1^ range.^27^ Reactive DCF metabolites can form adducts with liver proteins. However, these protein conjugates are not exclusively responsible for the toxic effects.^28,29^

Among pharmaceutical compounds, diclofenac is one of the most frequently analysed substances in the context of aquatic pollution. In European surface water and in drinking water, DCF concentrations of 1–7 ng L^−1^ have been found.^30^ In European sewage treatment plants, concentrations around 0.7 μg L^−1^ with a maximum of 11 μg L^−1^ were determined.^31^ 4′-OH-DCF was detected in Spanish sewage treatment plants in higher concentrations than DCF,^32^ but not in German sewage treatment plants.^33^ However, in the German sewage treatment plants, 4′-OH-DCF was the metabolite with the highest concentration.

DCF is subject to photodegradation, dependent on the irradiation time.^34–36^ Many degradation products, both metabolic and photochemical ones, have been structurally characterized.^34,37–39^ DCF tends to cyclise under specific conditions. The cyclisation product, DCF lactam (Fig. 1C), was also detected in sewage treatment plants.^33^ The photodegradation in surface water is compensated for by the permanent input of DCF from sewage treatment plants and leads to pseudo-constant concentrations that are subject only to seasonal fluctuations. For this reason, DCF had been included in the Watch List to Annex X of the Water Framework Directive of the European Union.^40^ DCF concentrations in European surface waters have been reported to exceed 100 ng L^−1^, which means above the PNEC (predicted no effect concentration) of 50 ng L^−1^. For Germany, concentrations of 2.55 μg L^−1^, which is higher than the annual average environmental quality standard (AA EQS) of 100 ng L^−1^, have been reported.^41^

Detection and quantification of DCF is performed by HPLC-MS, GC-MS, or immunoassays.^9,10,26,42,43^ There are many formats of immunoassays, for example ELISA,^1,3–10,26,43–45^ CLEIA,^46^ ULISA,^42^ ELIMSA,^47^ FLISA,^48^ FPIA,^49^ and LFIA.^50^ For DCF, the enzyme-linked immunosorbent assay (ELISA) is generally used in the indirect competitive format. A polyclonal^9^ antibody is commercially available and an excellent monoclonal antibody has been developed.^11^ Even earlier, in a medical study, monoclonal antibodies to DCF and its main metabolites, including 4′-OH-DCF, had been developed.^10^

These antibodies were obtained by immunization with immunogens in which DCF was directly conjugated to carrier proteins *via* its carboxyl function. The coating antigens used in ELISA studies were also based on DCF bound directly to the carrier protein. Other structures, that could possibly be used as haptens, are aceclofenac (ACF, see Fig. 1D), the glycolic acid ester of DCF, and DCF-Ahx (see Fig. 1E), an amide of 6-aminohexanoic acid and DCF. ACF is used as a prodrug and was hardly detected in sewage treatment plant samples, if at all, in a concentration of about 10 ng L^−1^.^33^

DCF-Ahx satisfies the rule for optimum spacer length of 6 C atoms.^51^ This substance was synthesized in our lab by solid-phase synthesis.^52^ The use of spacers between the analyte and the carrier protein helps in presenting the analyte to the antibody.

The aim of this study was the development of an ELISA based on the anti-DCF monoclonal antibody from the medical study (F01G21). The effect of a spacer between coating antigen und DCF on the sensitivity of all three anti-DCF antibodies was to be comparatively assessed. Finally, the application of the new ELISA (mAb F01G21, and employing a C_6_ spacer) to the analysis of sewage treatment plant samples and surface water samples is described. In addition, for the first time, photodegradation products have been isolated and studied as potential cross-reactants.

## Experimental

### Chemicals and equipment

Three anti-diclofenac antibodies were used: mAb 12G5 (ref. 10) was kindly provided by Prof. Dietmar Knopp, TU Munich, and mAb F01G21 (ref. 11) was provided by the co-authors from Salzburg. The polyclonal anti-DCF antibody pAb1 (ref. 9) was purchased (prod. no. ABIN289631, from rabbit, antibodies-online, Aachen, Germany). All solvents were chromatography grade. Diclofenac sodium salt and aceclofenac were purchased from Sigma-Aldrich, ^13^C_6_ labelled diclofenac sodium salt, 4′-hydroxydiclofenac and 1-(2,6-diclorophenyl)-2-indolinone from Fluka. Dimethylformamide (DMF), bovine serum albumin (BSA), ovalbumin (OVA), apotransferrin (APO) and protease from *Streptomyces griseus* were purchased from Sigma-Aldrich, too. Humic acid sodium salt (HA, 45–70%) was from Roth (Karlsruhe, Germany). For temperature control during some synthesis steps, an Eppendorf ThermoMixer® was used. HPLC: Ammonium acetate (NH_4_Ac, Fischer Chemicals, analytical grade, 99.3%), acetic acid glacial (AcOH, Fischer Chemicals, analytical reagent grade), methanol (MeOH, J.T. Baker, HPLC gradient grade), folded filters (qual., grade 1288, Sartorius Stedim, Göttingen, Germany), Strata™-X 33 μm polymeric reversed-phase SPE cartridges (500 mg, 6 mL, Phenomenex, Aschaffenburg, Germany). Instruments were a Milli-Q water purification system (Synthesis A10, Merck Millipore, Schwalbach, Germany) for ultrapure water, a microplate UV/Vis reader (SpectraMax® Plus 384, Molecular Devices, Biberach an der Riss, Germany), an automatic washer (BioTek ELx405 Select™, Bad Friedrichshall, Germany) and a plate shaker (Titramax 100, Heidolph, Schwabach, Germany). ELISA: Transparent 96 flat-bottom well microtiter plates with high binding capacity (Greiner Bio-One, Frickenhausen, Germany). Reagents: 1-*N*-hydroxysuccinimide (NHS), dicyclohexylcarbodiimide (DCC), trifluoroacetic acid (TFA), chlorotrimethylsilane, 3,3′,5,5′-tetramethylbenzidine (TMB, Serva, Heidelberg, Germany; research grade), Tween® 20 (Serva), sodium hydrogen carbonate (>98%, Fluka), sodium phosphate dibasic dihydrate (>99%), sodium chloride (>99.5%), sodium citrate monobasic (>99%), potassium sorbate (>99%), potassium phosphate dibasic (>99%), glycine (>99%), tris(hydroxymethyl)aminomethane (Tris, >99.8%) tetrabutylammonium borohydride (TBABH, >98%), *N*,*N*-dimethylacetamide (DMA, >99%), *N*,*N*-dimethylformamide (DMF, >99.8%), and casein were purchased from Sigma-Aldrich. For signalling, secondary antibodies labelled with HRP, secAb1-HRP (anti-mouse, A0168-1ML, Sigma-Aldrich) and secAb2-HRP (anti-rabbit, A6154, Sigma-Aldrich) were used.

### Coupling of diclofenac haptens

Haptens were conjugated to proteins *via* NHS/DCC method. The activation of the respective hapten (86.84 mM solution in dry DMF) was performed by adding 1.1 eq. NHS and 1.1 eq. DCC under argon (Ar) atmosphere. The reaction mixture was shaken at 750 rpm overnight (ON) in the dark at room temperature (RT). The side product *N*,*N*-dicyclohexylurea was separated by centrifugation at 4000 rpm at 4 °C for 10 min. After centrifugation, the supernatant solution was separated from the solid under Ar atmosphere. 60 μL of supernatant was added to 3 mg protein in 0.5 mL coupling buffer (0.13 M NaHCO_3_, pH 7.8). After 4 h reaction time in the dark at 22 °C in the ThermoMixer at 750 rpm, another 60 μL were added. After 4 h reaction time, the protein conjugate was purified by size exclusion chromatography on Sephadex™ G-25 in a PD-10 desalting column, PBS/water (1 : 9; v/v) being the eluent, collecting 96 fractions into a non-binding well plate. The coupling rate was determined by MALDI-ToF-MS as described.^44^

### Synthesis of DCF-Ahx-BSA

The C_6_ spacer conjugate DCF-Ahx-BSA was synthesized in a two-step NHS/DCC method. First, Boc-6-Ahx-BSA was produced (and lyophilized). After deprotection it was reacted with activated DCF. To produce Boc-6-Ahx-BSA, Boc-6-Ahx-OH (86.84 mM in dry DMF) was activated with 1.1 eq. NHS and 1.1 eq. DCC under Ar. The reaction mixture was stirred ON at RT. The side product *N*,*N*-dicyclohexylurea was separated by filtration under Ar. 1.8 mL of the filtrate were added to 200 mg BSA in 40 mL coupling buffer (0.13 M NaHCO_3_, pH 7.8). The reaction mixture was stirred for 4 h at RT. After this, another 1.8 mL of the activated and protected spacer derivate were added, the reaction mixture stirred (ON, RT). The protein was purified by centrifugation and washing five times with water in an Amicon® filter unit (30 min, 4000 rpm). After washing, the protein was dissolved in water and lyophilized to a white solid (ON). For the second step, Boc-6-Ahx-BSA was dissolved in TFA (ON). TFA was removed under vacuum. The deprotected protein was purified by centrifugation and washing five times with water in an Amicon® filter unit (30 min, 4000 rpm). The protein was then dissolved in water and lyophilized to a white solid ON. Diclofenac (86.84 mM) was activated in dry DMF with 1.1 eq. NHS and 1.1 eq. DCC under Ar atmosphere. The reaction mixture was stirred ON at RT. On the next day, the side product was separated by filtration under Ar. The white protein solid was dissolved in 35 mL coupling buffer (0.13 M NaHCO_3_, pH 7.8). 1.8 mL of the activated DCF were added to the protein solution. The reaction mixture was stirred for 4 h at RT. Hereupon, another 1.8 mL activated DCF were added, and the reaction mixture stirred ON at RT. On the following day, the protein was purified by centrifugation and washed with water in an Amicon® filter unit for 30 min with 4000 rpm per washing step. The protein was washed five times. After washing, the protein was dissolved in water and lyophilized to a white solid. The success of coupling was determined by MALDI-ToF-MS, indirect competitive ELISA and HPLC analysis after proteolytic digestion.

### Proteolytic digestion

An amount of 2 mg DCF-6-Ahx-BSA was dissolved in 1 mL water. 1 mg of the protease from *Streptomyces griseus* was added. The reaction mixture was shaken ON in a ThermoMixer at 750 rpm and 37 °C. On the following day, the solution was filtered through a 0.45 μm syringe filter. The filtered solution was analysed by HPLC-MS.

### General protocol of the indirect competitive ELISA

The ELISA was carried out in microtiter plates with high protein binding capacity. The surface of every well of the plates was coated by adding 200 μL of the respective coating antigen, always diluted in PBS (1.56 g L^−1^ NaH_2_PO_4_ × 2H_2_O, 12.46 g L^−1^ Na_2_HPO_4_ × 2H_2_O, 8.47 g L^−1^ NaCl, pH = 7.6) but in different dilutions, and incubating for 18 h. Every incubation step was done on a plate shaker at 750 rpm and RT. After automatic washing with 1 : 60 diluted washing buffer (6.12 g L^−1^ KH_2_PO_4_, 65.31 g L^−1^ K_2_HPO_4_, 225 mg L^−1^ C_6_H_7_KO_2_, 30 mL L^−1^ Tween™ 20, pH = 7.6), unoccupied areas were blocked with 200 μL 0.1% casein in PBS for 1 h. After washing, 150 μL of DCF calibrators in water and 50 μL of different dilutions of the respective antibody in Tris buffer (1.21 g L^−1^ Tris, 8.77 g L^−1^ NaCl, pH = 8.5) were pipetted into each well. After 1 h incubation and a washing step, 200 μL of different dilutions of HRP-labelled, secondary antibody (anti-mouse/anti-rabbit) were added. After 1 h incubation, the final washing step was carried out. 200 μL of a substrate solution (8.1 μL H_2_O_2_ and 525 μL TMB in 21 mL citrate buffer (47.10 g L^−1^ C_6_H_7_NaO_7_, pH = 4.0)) were added to the empty wells and substrate turnover given 30 min. It was stopped by adding 100 μL H_2_SO_4_ (1 M). Absorbance was determined on a microplate reader at 450 nm and referenced to 620 nm. All calibrators were determined in triplicate. SoftMax® Pro Software (v.5.3, Molecular Devices) was used for data acquisition. Sigmoidal standard curves were obtained by fitting a four-parameter logistic function to the data points. The parameter for maximum signal (upper asymptote of the calibration curve) and the parameter that reflects the concentration at the inflection point of the curve (sometimes: test midpoint, ≈IC_50_) were used for ELISA performance comparison.

### Final protocol of the indirect competitive ELISA

The surface of the wells was coated by adding 200 μL ACF-APO (2.3 mg mL^−1^), diluted 1 : 50 000 in PBS. After washing, blocking was done with 200 μL 0.1% casein in PBS for 1 h. After washing, 150 μL of DCF calibrators in water and 50 μL anti-DCF antibody, diluted 1 : 10 000 in Tris buffer or “sample buffer” (36.34 g L^−1^ Tris, 26.30 g L^−1^ NaCl, and 11.94 g L^−1^ Na_2_EDTA × 2H_2_O, pH = 7.6), diluted 1 : 1, were pipetted into each well. After 1 h incubation and a washing step, 200 μL HRP-labelled secondary antibody, diluted 1 : 50 000 in PBS was added, and after 1 h incubation another washing performed. 200 μL of the substrate solution was added, and colour development occur for 30 min before stopping by 100 μL H_2_SO_4_ (1 M).

### Determination of cross-reactivities

To determine the selectivity of the ELISA, potential cross-reactive substances, *i.e.*, structurally related metabolites and photodegradation products, were assayed using the final protocol. Stock solutions with concentrations of 1000 mg L^−1^ were prepared in MeOH. Dilutions (concentrations between 10 mg L^−1^ and 0.1 ng L^−1^) were prepared in water by serial dilution. The IC_50_ values of the substances (molar concentration giving 50% inhibition) are expressed as percent relative to the IC_50_ value of DCF.

### Assessment of matrix effects

The influence of organic solvents was tested with standards containing 1%, 2%, 2.5%, 5%, 10%, 20%, 25%, 30%, 40% and 50% of methanol, ethanol, acetonitrile, DMSO, DMF, isopropanol, or acetone. For testing of the influence of natural organic matter (NOM), NOM from the lakes “Grosse Fuchskuhle” and “Schwarzer See”, collected in the summer of 2003, was added to the calibrators in concentrations of 1.25 mg L^−1^, 2.5 mg L^−1^, 5 mg L^−1^, 10 mg L^−1^, and 50 mg L^−1^. The elemental composition of NOM^53^ is listed in the ESI.†

Humic acid (HA) was added to calibrators, too, in concentrations of 0.1 mg L^−1^, 0.2 mg L^−1^, 0.5 mg L^−1^, 1 mg L^−1^, 1.25 mg L^−1^, 2.5 mg L^−1^, 5 mg L^−1^, 10 mg L^−1^ and 50 mg L^−1^. The influence of salinity was tested in the range of 5–30 g L^−1^ NaCl, and 50 mg L^−1^ and 75 mg L^−1^ MgCl_2_, respectively.

### Solid-phase extraction (SPE)

SPE was performed on an AutoTrace™ SPE workstation (Thermo Scientific Dionex, Idstein, Germany). First, Strata™-X cartridges were washed with 10 mL MeOH, equilibrated with 10 mL ultrapure water, followed by loading the respective sample volume of 1000 mL. The flow rate for each step was 10 mL min^−1^. Afterwards, the cartridges were dried by flushing N_2_ (20 psi) for 15 min through the columns and the adsorbates were eluted with 10 mL MeOH. The solvent of the eluate was evaporated under a flow of N_2_. The residue was taken up with ultrapure water and filtered through a syringe glass fibre filter (no. 7-8808, neoLab, Heidelberg, Germany). After filtration, the samples were stored at 4 °C.

### Synthesis of DCF methyl ester (DCF-Me)

1 g DCF was dissolved in 7 mL DMF. After addition of 7 mL methyl iodide, the reaction mixture was stirred ON. On the next day, the solvent was removed under vacuum. The solid was suspended in ethyl acetate. The organic solution was washed with water three times. The organic phase was dried with magnesium sulfate. After filtration, ethyl acetate was removed under vacuum. A yellow solid of 893 mg (90% yield) was obtained.

Identity was confirmed by the mass spectrum (LC-MS method) of the compound, with the base peak at 311.96 Da (DCF-Me), see Fig. S11 (ESI†).

### Synthesis of 4′-OH-DCFMe

An amount of 767 μg 4′-OH-DCF was dissolved in 100 μL chlorotrimethylsilane. After addition of 400 μL MeOH, the reaction mixture was shaken overnight. On the following day, the solvent was removed under vacuum. A white solid was obtained.

### Isolation of photodegradation products

A saturated solution of DCF in water was irradiated with a Vilber UV lamp VL-6.C with a wavelength of 254 nm and a power of 12 W for 4 days, 8 hours per day. After this time, the water was removed under a stream of air. The crude product was dissolved in EtOAc/*n*-hexane (1 : 1, v/v) and purified *via* column chromatography. Two photodegradation products were isolated. One structure was confirmed by NMR (data not provided).

In another approach, a DCF solution was electrochemically oxidized under conditions published by Faber *et al.* (2012).^38^ The structures could be confirmed by their mass spectra, compared to literature.^37^ Cross-reactivity studies of the oxidized products were done by LC-ELISA.^54^

### LC-MS analysis

HPLC-MS runs were performed on an Agilent 1260 Infinity LC system with binary pump, degasser, autosampler, column heater, and UV detector. The chromatographic separation was carried out on a Kinetex X_B_-C18, 2.6 μm, 150 mm × 3 mm analytical LC column with a UHPLC C18, 3 mm guard column (both from Phenomenex). As mobile phases, ultrapure water with 10 mM NH_4_Ac and 0.1% (v/v) acetic acid (A) and methanol with 10 mM NH_4_Ac and 0.1% (v/v) acetic acid (B) were used. Flow rate was 350 μL min^−1^, column heated to 50 °C. An elution gradient was applied, starting with 20% B for the first three minutes. Within five minutes, B is ramped to 95% and maintained at this level for another four minutes. Then B is reduced to 20% within two minutes and held for eight minutes to re-equilibrate the column. Injection volume was 10 μL. Mass analysis was performed on an AB SCIEX 6500 triple-quad mass spectrometer (SCIEX, Darmstadt, Germany) with electrospray ionization (ESI), in positive ionization mode.

### LC-MS/MS

The same system as with LC-MS analysis was used. The mobile phases in this case were ultrapure water with 0.1% (v/v) acetic acid (A) and MeOH with 0.1% (v/v) acetic acid (B). The flow rate was 350 μL min^−1^ and the column heater temperature was 55 °C. An elution gradient was applied, starting with 30% B, held for 3 min. Afterwards B was ramped to 95% within 11 min and held constant for the next 4 min, decreased back to 30% B within 0.5 min and held for the next 7.5 min to re-equilibrate the column. The injection volume was 10 μL, too. Parameters used to produce fragment ions in selected reaction monitoring mode (SRM) and collision energies (CE) are given in Table 1. The electrospray ionization source (ESI) was operated in the positive ionization mode.

**Table 1:** Selected reaction monitoring (SRM) mass transitions and the collision energies for diclofenac and its metabolites and the ^13^C internal standard

Compound	SRM transition	CE (V)
DCF	296 → 250 quantifier	22
	296 → 214 qualifier	30
^13^C_6_-DCF	302 → 256 quantifier	22
	302 → 220 qualifier	30
Hydroxy-DCF	312 → 266 quantifier	22
	312 → 230 qualifier	30
Cyclo-DCF	278 → 151 quantifier	70
	278 → 214 qualifier	40
	278 → 179 qualifier	60
^13^C_6_-Cyclo-DCF	284 → 157 quantifier	70
	284 → 220 quantifier	40
	284 → 185 quantifier	60
Aceclofenac	354 → 250 quantifier	22
	354 → 214 qualifier	30

The parameters used for ionization were a temperature of 400 °C, 4500 V ion spray voltage, an entrance potential (EP) of 10 V, a declustering potential (DP) of 90 V, a collision cell exit potentials (CXP) of 15 V, a curtain gas with 35 psi (1 psi = 0.0689 bar = 6890 kg m^−1^ s^−2^ (SI unit)), a nebulizer gas (GS1) with 62 psi, a turbo gas (GS2) with 62 psi and a collision gas with 8 psi. Analyst® version 1.6.2 software (SCIEX) was used to control the instrument, acquire data and evaluate the results.

### LC-ELISA

LC-ELISA coupling was performed as described before for estrone.^54^ The electrochemically generated oxidized products of DCF were separated by HPLC and fractions collected. A 200 μL sample of each fraction was pipetted into transparent, untreated 96-well flat-bottom microtiter plates (Nunc™ Thermo Fisher). The solvent was removed in an air flow. After re-dissolving in 200 μL ultrapure water and shaking for 15 min, a 150 μL sample was transferred to a coated and blocked microtiter plate prepared according to the final ELISA protocol. An amount of 50 μL antibody in a 1 : 10 000 dilution was added to each well and the ELISA performed as described in the final protocol.

## Results and discussion

### Synthesis of new protein conjugates

A “C_6_ spacer version” of DCF (DCF-6-Ahx), DCF extended by a 6-aminohexanoic acid (6-Ahx) spacer, was previously produced.^52^ In this work, it was coupled to ovalbumin (OVA) and apotransferrin (APO). Commercially available aceclofenac (ACF), which can be considered a “C_2_ spacer version” of DCF, was conjugated with APO only. For comparison, a DCF-OVA conjugate was also employed.

An inverse concept for hapten binding to a protein was developed in parallel, creating a “spacer derivative” of the protein first. For this, Boc-protected 6-aminohexanoic acid (Boc-6-Ahx-OH) was coupled to the most accessible lysine (Lys) residues of bovine serum albumin (BSA) after NHS activation, to yield Boc-6-Ahx-BSA. Purification was achieved *via* an Amicon® filter unit, Boc cleaved in TFA. This strategy is also applicable to APO, but not to larger proteins such as keyhole limpet hemocyanin (KLH), which is also rather costly to be applied in synthesis, or thyroglobulin (TG) which is not soluble in TFA. The use of Fmoc-protected 6-aminohexanoic acid (Fmoc-Ahx-OH) is unsuitable since Fmoc cleavage requires basic conditions which would destroy the proteins. After Boc cleavage and purification, NHS-activated DCF could be coupled to the spacer derivative of BSA (6-Ahx-BSA). The result is BSA, to which DCF is bound with an inserted C_6_ spacer – without synthesising an analyte spacer derivative. The success of this method was shown by proteolytic digestion and HPLC-MS analysis and by ELISA using DCF-6-Ahx-BSA as coating reagent. All details are given in the ESI (Fig. S1–S3†). The concentrations and coupling densities of the produced conjugates are shown in Table 2.

**Table 2:** Concentration and mean coupling density of protein conjugates

Conjugate	Concentration [mg mL^−1^]	Mean coupling density
DCF-OVA	2.0	3.0
DCF-6-Ahx-OVA	0.8	2.9
DCF-6-Ahx-APO	1.5	28
ACF-APO	2.3	25
DCF-6-Ahx-BSA	0.4	22
Boc-6-Ahx-BSA	6.1	46

### ELISA optimization and comparison of the coating antigens

ELISA optimization was carried out using a checkerboard approach. Several plates were coated with the respective coating antigens in various dilutions. In the absence of the analyte, the respective antibody (pAb1, mAb 12G5, or mAb F01G21) was added in various dilutions. The respective HRP-labelled secondary antibodies (secAb1-HRP, secAb2-HRP) were also tested in various dilutions. Calibration curves were recorded and compared for those with maximum absorbances between 0.5 and 1.

**Fig. 2:**
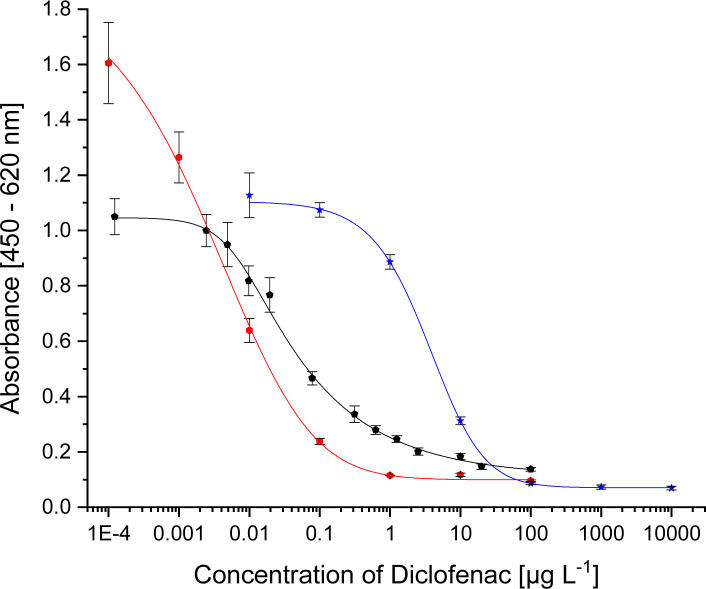
Calibration curves for <svg xmlns="http://www.w3.org/2000/svg" version="1.0" width="18.545455pt" height="16.000000pt" viewBox="0 0 18.545455 16.000000" preserveAspectRatio="xMidYMid meet"><metadata> Created by potrace 1.16, written by Peter Selinger 2001-2019 </metadata><g transform="translate(1.000000,15.000000) scale(0.015909,-0.015909)" fill="currentColor" stroke="none"><path d="M400 760 l0 -40 -40 0 -40 0 0 -40 0 -40 -80 0 -80 0 0 -40 0 -40 -40 0 -40 0 0 -40 0 -40 40 0 40 0 0 -40 0 -40 -40 0 -40 0 0 -40 0 -40 40 0 40 0 0 -80 0 -80 40 0 40 0 0 -80 0 -80 280 0 280 0 0 40 0 40 40 0 40 0 0 160 0 160 40 0 40 0 0 80 0 80 -40 0 -40 0 0 40 0 40 -40 0 -40 0 0 40 0 40 -80 0 -80 0 0 40 0 40 -120 0 -120 0 0 -40z"/></g></svg> pAb1 (1 : 32 000, coating with DCF-6-Ahx-OVA 1 : 40 000, secAb2-HRP 1 : 40 000) 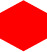 mAb 12G5 (1 : 10 000, coating with DCF-6-Ahx-Apo 1 : 50 000, secAb1-HRP 1 : 20 000) and 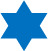 mAb F01G21 (1 : 10 000, coating with ACF-Apo 1 : 50 000, secAb1-HRP 1 : 40 000).

For pAb1, the novel coating antigen DCF-6-Ahx-OVA proved to be most compatible. For mAb 12G5, DCF-6-Ahx-APO was determined to be the best coating antigen. The new conjugates decrease the test midpoints in comparison to the published values. All three optimized systems are shown in Fig. 2. The test midpoints and the published values are listed in Table 3. The comparisons of the conjugates with the different protein conjugates are shown in the ESI (Fig. S4A–C†).

**Table 3:** Test midpoints of the indirect competitive ELISAs

Antibody	Coating antigen	IC_50_	Published IC_50_
pAb1	DCF-6-Ahx-OVA	30 ng L^−1^	60 ng L^−1^ ^9^
mAb 12G5	DCF-6-Ahx-Apo	9 ng L^−1^	44 ng L^−1^ ^10^
mAb F01G21	ACF-APO	3 μg L^−1^	

For mAb F01G21 the conjugate ACF-APO, with a shorter spacer, proved to be the best coating antigen paying tribute to the rule that hapten selection should be done for each antibody individually.

### Consolidated measurement range of the ELISA with antibody mAb F01G21

**Fig. 3:**
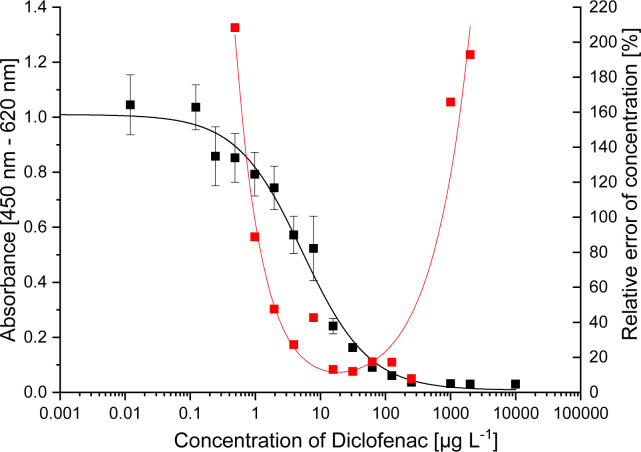
Precision profile showing the relative error of DCF concentration estimates (■ calibration, 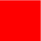 relative error).

After this optimization, a precision profile, which is shown in Fig. 3, was recorded, which takes into account both the intra- and interplate reproducibility as well as the reproducibility on different days of a week, during which dilutions were repeatedly re-established. The analytical range is defined as the range with a relative error of concentration lower than 30%. This corresponds to DCF concentrations of 2–100 μg L^−1^. The ELISA is therefore suitable for applications in sewage treatment plants. Drinking water and surface water samples can be measured after pre-concentrating by SPE.

### Solvent influence

**Fig. 4:**
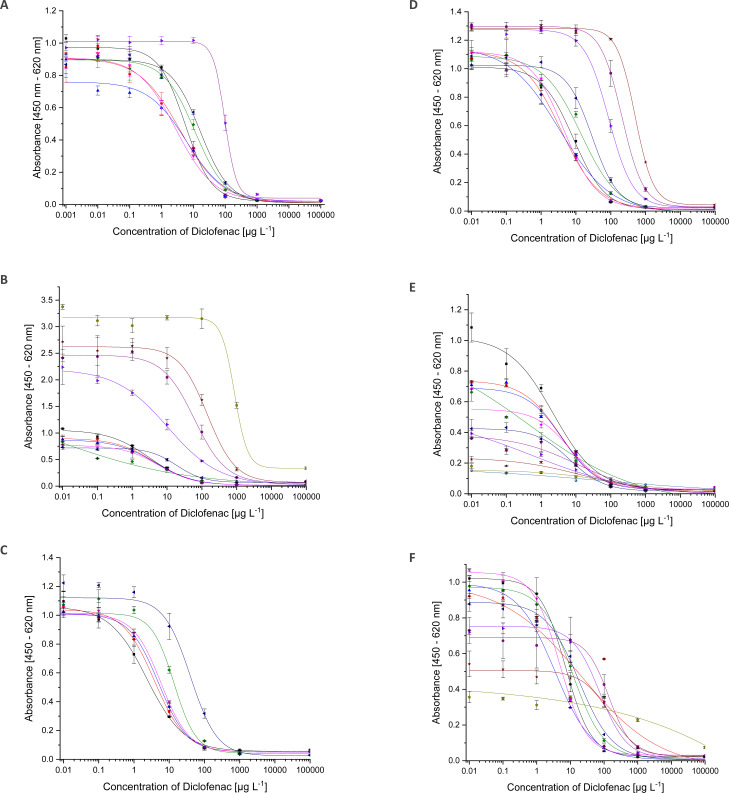
ELISA calibration curves for DCF with increasing solvent content ((A) methanol, (B) ethanol, (C) acetonitrile, (D) isopropanol, E DMSO, F DMF, ■ 0%, 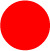 1%, 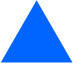 2%, 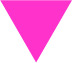 2.5%, 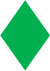 5%, 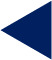 10%, 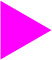 20%, 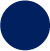 25%, 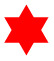 30%, 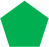 40%, 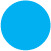 50%).

In many cases, a pre-concentration of the analyte by means of SPE is carried out before analysis. There, the retained analyte is eluted with organic solvents from the cartridge. In most cases, methanol (MeOH) is used for this purpose. Other solvents are used for sample extraction, *e.g.*, from soil. With solvents, there is danger of denaturing the antibody. In addition, free DCF, coated DCF and antibody-bound DCF (from the sample or from the surface of the microtiter plate) are in an equilibrium during the indirect ELISA. Equilibria are dependent on the solvent composition as one aspect. Thus, the equilibrium can shift with increasing methanol content. As can be seen in Fig. 4A, the maximum absorbance shows slight fluctuations. The changes in the IC_50_ are far more relevant. No significant changes occur below a share of 5% MeOH. The test midpoint is doubled with 5% MeOH, and it triples with 10% MeOH. With 20% MeOH, the curve changes drastically. From 25% MeOH on, a reliable curve is no longer visible. Therefore, the methanol content in the samples should be less than 10%. Accordingly, it is advisable, that samples be completely dried after solid-phase extraction and then taken up in ultrapure water.

For ethanol (EtOH) (Fig. 4B), the situation is the same. Up to 5% EtOH, the curve is almost identical, except for slight fluctuations of the upper asymptote. With a solvent content of 10% ethanol, the test midpoint increases by a factor of four. A significantly different course of the curve could only be observed from 20% ethanol onwards. The ethanol content of a sample should not exceed 5%.

In the case of an acetonitrile (ACN)/water mixture (Fig. 4C), all the curves run identically to about 2.5% solvent content. Above 5%, the curve changes significantly. Consequently, samples cannot be analysed correctly.

The same behaviour as for ethanol was observed for isopropanol (iPrOH), which is shown in Fig. 4D.

The most stable system is a mixture of water and dimethylsulfoxide (DMSO). The results are shown in Fig. 4E. Even with a 50 : 50 mixture, an ELISA can be carried out, which was not possible with any other solvent-water mixture. However, the values of maximum absorbance decrease continuously. Already with 1% DMSO, the parameter A has decreased so much that comparable quantification is no longer possible. The test midpoint has doubled from 2% DMSO on. Samples should therefore not contain DMSO. Fortunately, it is almost not used in sample processing techniques.

Dimethylformamide (DMF) has no influence on the ELISA curve up to 5%. As can be seen in Fig. 4F, deviations start at 10%.

Up to 2.5%, acetone is compatible with the ELISA. Deviation in the curve occurs at 5% (curves not shown).

**Fig. 5:**
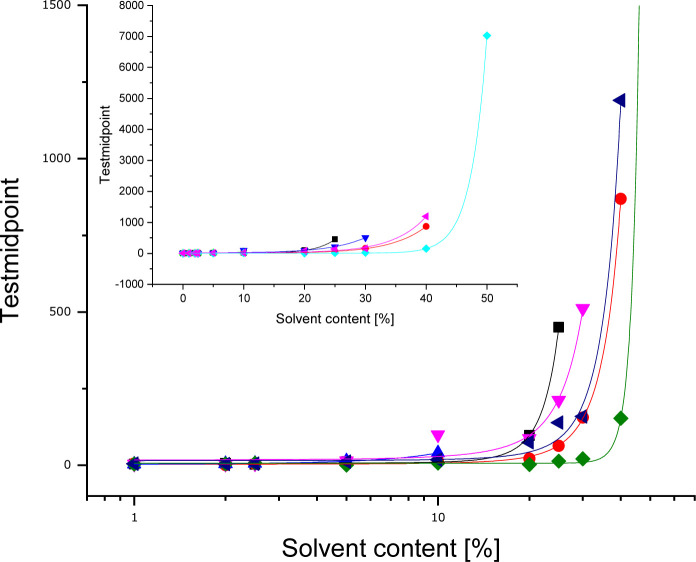
ELISA test midpoints as a function of the solvent fraction (■ methanol, 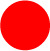 ethanol, 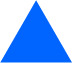 acetonitrile, 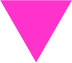 isopropanol, 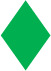 DMSO, 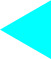 DMF).

In the ESI† all curves are shown in 3D plots (Fig. S5†). The test midpoints (≈IC_50_) of the curves have been connected to illustrate trends. For all solvents, parameter C can be correlated with the solvent content. The dependency follows an exponential function. Additional to the fact, at which solvent content the ELISA system is disturbed, the correlated data (see Fig. 5) allows to derive the stability of mAb F01G21 for organic solvents. It is in the following order of decreasing stability: DMSO > EtOH > DMF > iPrOH > MeOH > ACN.

### Establishment of a sample buffer

**Fig. 6:**
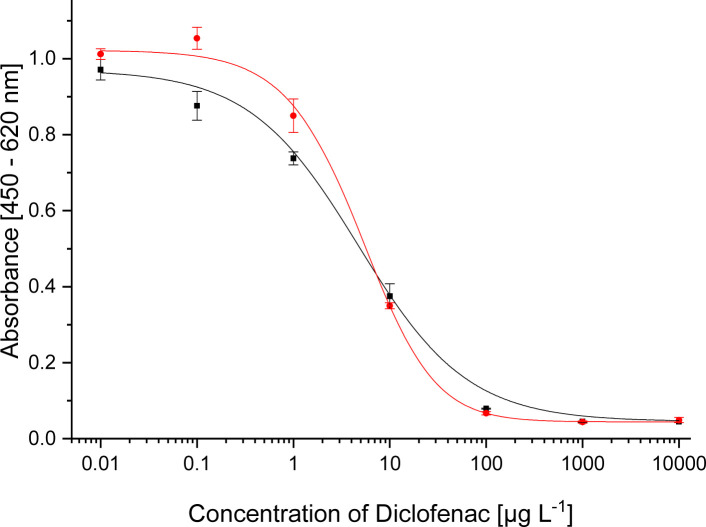
Comparison of DCF calibration functions, using two different buffers for antibody dilution (■ Tris, 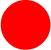 sample buffer).

In earlier work,^11^ mAb F01G21 had been diluted in Tris buffer. For the optimisation work, ELISA curves were obtained *via* DCF calibrators in ultrapure water. However, real samples contain a variety of possible components, for example salts or organic compounds. Therefore, the use of a “sample buffer” to dilute mAb F01G21 seemed useful. The buffer of choice was a concentrated Tris buffer with pH = 7.6 that also contained disodium ethylenediamine tetraacetate (Na_2_EDTA). EDTA is known as complexing agent. Before diluting the antibody, the buffer must be diluted 1 : 1 in ultrapure water. In combination with the sample, the buffer reaches its final (8-fold Tris) concentration, assuring safe buffering of the sample pH and an overall high salt concentration in all samples that should stabilize the antibody. The calibration curves recorded in Tris buffer and sample buffer, respectively (Fig. 6), show only small deviations, insignificant when samples and calibrators are both diluted in this specific sample buffer.

### Influence of sample salinity

**Fig. 7:**
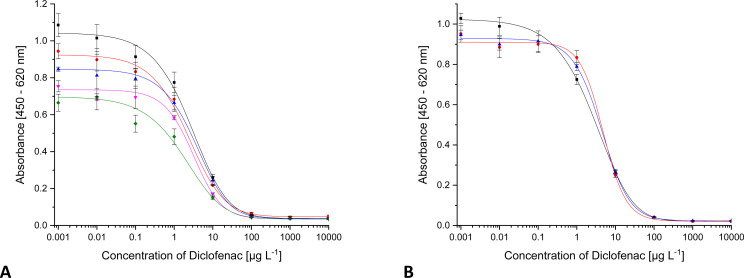
ELISA calibration function for DCF with increasing concentration of different salts ((A) ■ 0 g L^−1^ NaCl, 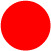 5.8 g L^−1^ NaCl, 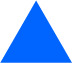 10 g L^−1^ NaCl, 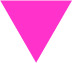 20 g L^−1^ NaCl, 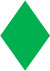 30 g L^−1^ NaCl; (B) ■ 0 mg L^−1^ CaCl_2_, 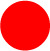 50 mg L^−1^ CaCl_2_, 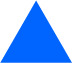 75 mg L^−1^ CaCl_2_).

An important disturbing component can be the salt concentration of a sample. *Via* DCF calibrators in ultrapure water, spiked with defined salt concentrations, this influence was studied. The tested salt concentrations have no influence on the test midpoint. However, they lead to a decrease of the signal in the lowest concentration ranges. 6 g L^−1^ NaCl leads to a decrease of the signal intensity by 10%, shown in Fig. 7A.

However, this still lies within the range of tolerable fluctuations that can occur within the standard deviations of individual values. An amount of 30 g L^−1^ causes a signal drop by 36%. However, such a salt concentration can only be found in seawater samples. Surface waters and drinking water contain only traces of sodium chloride, which do not influence the ELISA.

The experiments probing the influence of the chaotropic ion calcium (Ca^2+^) are depicted in Fig. 7B. Even at the highest concentration of 75 mg L^−1^, no change in the calibration function could be found. In drinking water and surface waters salt concentrations are in the low mg L^−1^ range. In summary, no influence of salinity on ELISA measurements in environmental samples is to be expected.

### pH stability

**Fig. 8:**
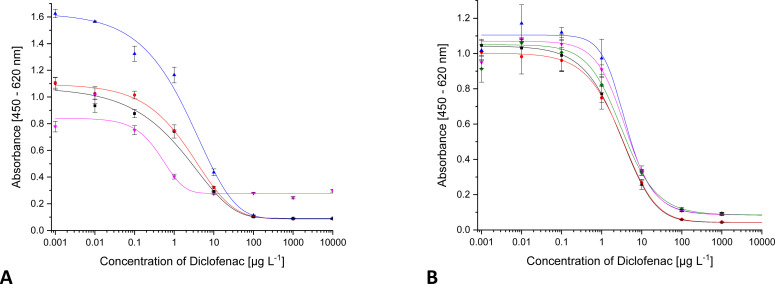
pH dependency of the DCF calibration function: (A) ■ neutral pH, 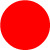 pH = 3, 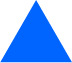 pH = 2, 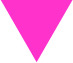 pH = 1 (B) ■ neutral pH, 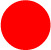 pH = 9, 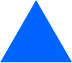 pH = 10, 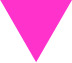 pH = 11, 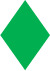 pH = 12.

Several sample preparation procedures require acidic or basic conditions. The pH value tolerated by an antibody in the ELISA depends on the buffer capacity. In order to test the tolerance range, DCF standard solutions were adjusted to pH values of 1–12 using HCl and NaOH, and measured. The acidic range is shown in Fig. 8A. Identical results from neutral down to pH = 3 can be achieved. Lower pH values alter the maximum signal or the shape of the curve, which is because antibodies have a pH optimum in the physiological range around 7.6. This pH can no longer be maintained by the buffer. According to these results, it is theoretically possible to analyse samples with pH values of 1 or 2 using corresponding (“matrix-matched”) calibrator series. The results, however, could be questionable since DCF is known to cyclize under acidic conditions. Since, as described later, the resulting DCF-lactam shows a cross-reactivity of <0.01%, the measured DCF concentrations would be too low, decreasing with time.

In the basic area, displayed in Fig. 8B, there is no restriction. Even at pH 12, the curve is quite comparable to that of the neutral standards. Since proteins (the antibodies) are hydrolysed under basic conditions but stable under acidic ones, this shows that the antibody is protected from basic conditions by the sample buffer.

### Matrix effects

**Fig. 9:**
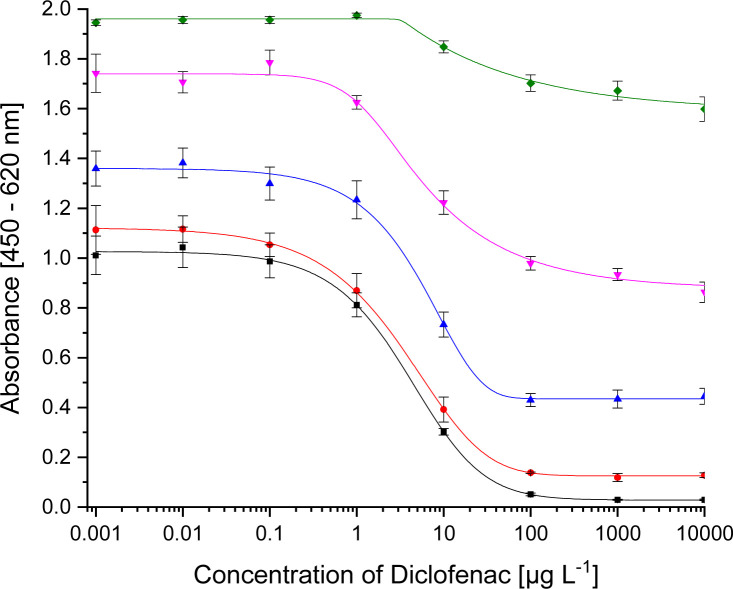
ELISA calibration function for DCF with increasing humic acid concentration (■ 0 mg L^−1^ HA, 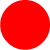 2.5 mg L^−1^ HA, 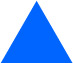 5 mg L^−1^ HA, 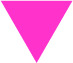 10 mg L^−1^ HA, 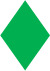 20 mg L^−1^ HA).

The most important factor to be assessed in antibody characterisation for environmental analysis is the organic matrix. Matrix components can interact in a variety of ways with the components of an immunoassay. Some matrix compounds may even be pre-concentrated together with the analyte during SPE. The organic matrix consists of a multitude of different compounds that have no or only a rough common structure. Humic acids (HA) are often used to assess the effect. So, DCF standards were spiked with different humic acid concentrations and measured. The results are shown in Fig. 9. Up to 2.5 mg L^−1^ of humic acid, all curves are quite similar. Above 2.5 mg L^−1^, an increase of the maximum signal as well as the signal background (base signal) occurs. From 20 mg L^−1^ on, no curve is obtained. This may be an effect of non-specific binding. Humic acid in water samples is present as a poly-anion and could bind to proteins *via* electrostatic interactions. This would lead to an accumulation of the antibody on the microtiter plate and to an overestimation of the analyte. Such an effect has already been observed with other ELISAs for DCF.^9^

**Fig. 10:**
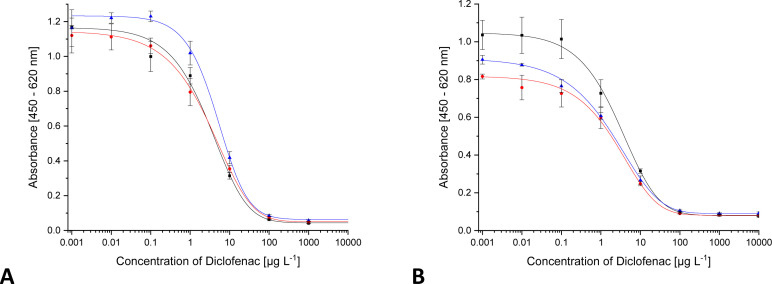
ELISA calibration function for DCF with increasing content of NOM ((A) NOM isolated from Groβe Fuchskuhle; ■ 0 mg L^−1^ NOM, 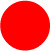 1.25 mg L^−1^ NOM, 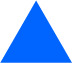 2.5 mg L^−1^ NOM; (B) NOM isolated from Schwarzer see; ■ 5 mg L^−1^ NOM, 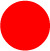 10 mg L^−1^ NOM, 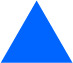 50 mg L^−1^ NOM).

In a natural aqueous system, humic acid is only one component of the organic matrix besides many others, albeit with the highest share. For this reason, additionally, the influence of organic matrix was investigated adding to the calibrators natural organic matter (NOM), isolated from the lake Groβe Fuchskuhle. The total organic carbon content (TOC) in Groβe Fuchskuhle is 343.7 mg g^−1^ (mg carbon per g NOM). Thus, in the calibrators with 50 mg L^−1^ NOM, an organic carbon concentration of 17.2 mg L^−1^ is present. No big changes in the calibration curves were observed (Fig. 10A).

Because the nature of NOM is different for each water, the same experiment was performed with NOM isolated from the lake Schwarzer See. As seen in Fig. 10B from 10 mg L^−1^ NOM on, there is a slight deviation of the curve. In Schwarzer See, the TOC is 210.4 mg g^−1^ and is thus lower than in Groβe Fuchskuhle. The maximum concentration of NOM corresponds to a TOC content of 10 mg L^−1^. The deviation in the curve was observed at 2.1 mg L^−1^, a much lower carbon content than in Groβe Fuchskuhle.

**Fig. 11:**
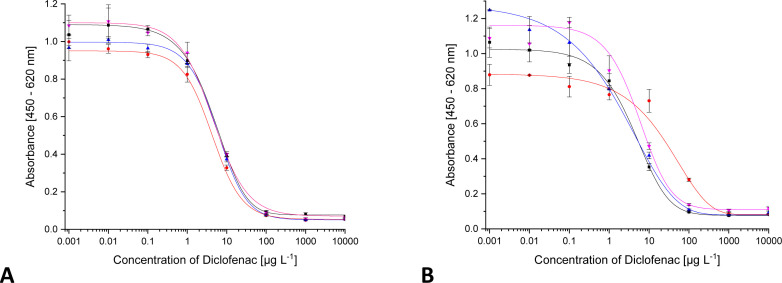
ELISA calibration function for DCF in different water samples ((A) without SPE, ■ Millipore-Q water, 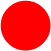 drinking water, 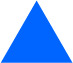 tap water, 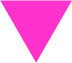 garden pond; (B) SPE pre-concentrated 1 : 600, ■ Millipore-Q water, 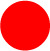 drinking water, 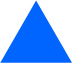 garden pond, 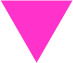 Flakensee).

In order to study the influence of matrix components in the real system, drinking water and tap water samples, as well as a sample from a private garden pond with fish and aquatic plants were spiked with DCF. Before, the absence of DCF in the samples was checked by HPLC-MS/MS. The acquired calibration curves (see Fig. 11A) were compared with the standard curve from calibrators in ultrapure water: Basically, all curves are the same. The matrix components of these typical real-word samples had no influence on the ELISA.

To improve sensitivity, real-world samples may be subjected to SPE. In order to investigate the influence of pre-concentrated matrix, the drinking water and the garden pond sample, as well as a sample collected from lake Flakensee (absence of DCF confirmed by HPLC-MS/MS), were pre-concentrated 1 : 600 *via* SPE. The concentrates were used to prepare DCF calibrators. The ELISA results (Fig. 11B) were compared with the reference in ultrapure water: Only slight fluctuations at low concentration were found, which correspond to ng L^−1^ concentrations in drinking water or surface water samples. Summarizing, matrix effects of real-world samples on mAb F01G21 were rather small.

### Cross-reactivities

Cross-reactivity studies are used for the estimation of the selectivity of an antibody. Cross-reactivity values are calculated from the ratios of the test midpoints (IC_50_) of the respective ELISA calibration curves with the target analyte and the cross-reactants, as shown in eqn (1).1
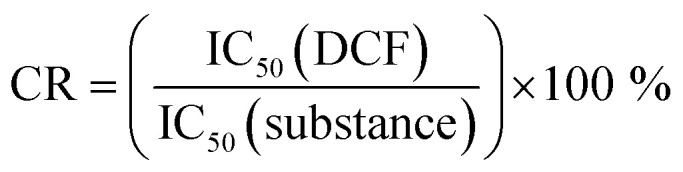


Eqn (1) Calculation of cross-reactivity (CR).

Various contaminants which may be present in environmental samples, such as caffeine, carbamazepine, bisphenol A, cetirizine, cocaine, isolithocholic acid, and sulfamethoxazole, were tested in the assay with antibody mAb F01G21. No cross-reactivity could be detected up to concentrations of 10 mg L^−1^. The same applies to the fragments of diclofenac, aniline, 2,6-dichloroaniline, and phenylacetic acid. DCF metabolites, *e.g.*, the glucuronide of diclofenac and structurally related compounds, which are also used as pharmaceuticals, as well as the methyl ester of DCF (DCF-Me), with specific uses,^55^ were assayed, too (Table 4).

**Table 4:** Cross-reactivities of mAb F01G21 and threshold values for overestimation. Threshold values were determined by quantifying DCF standards with cross-reactants of various concentrations. Above the threshold values, the results are no longer identical with those in the absence of the cross-reactant

Substance	Structure
Cross-reactivity	
Threshold value	
ACF	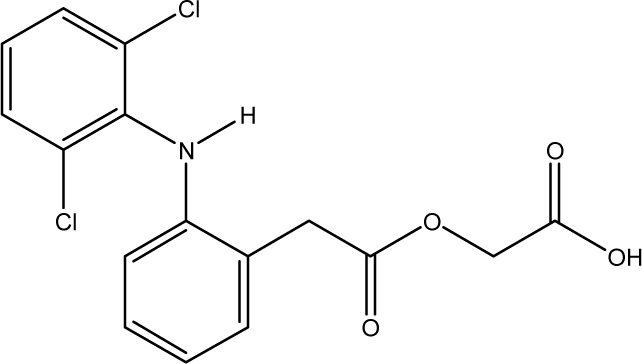
61%	
10 μg L^−1^	
DCF-Ahx	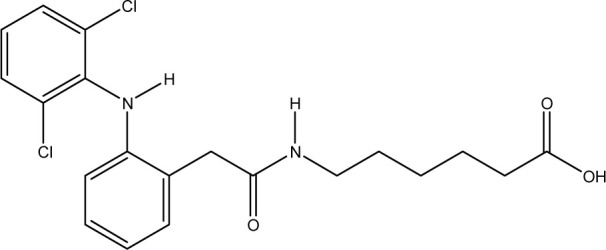
150%	
1 μgL ^−1^	
DCF-Me	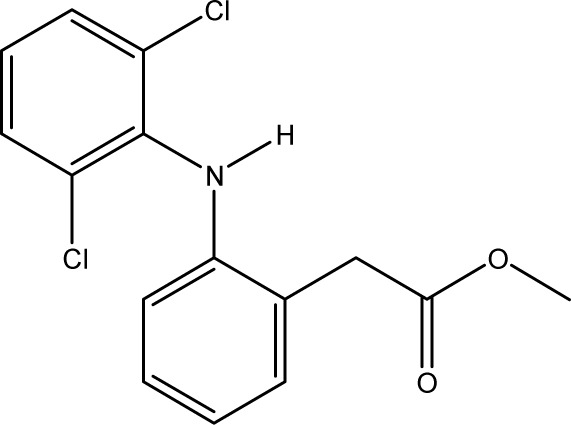
44%	
1 μg L^−1^	
DCF-glucuronide	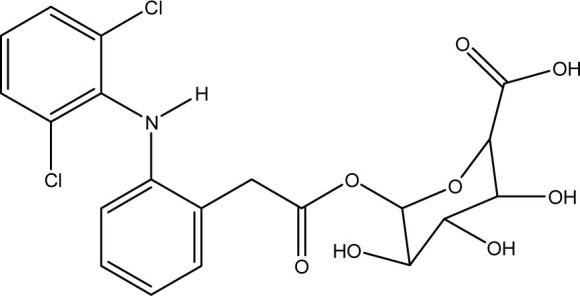
57%	
1 μg L^−1^	
4′-OH-DCF	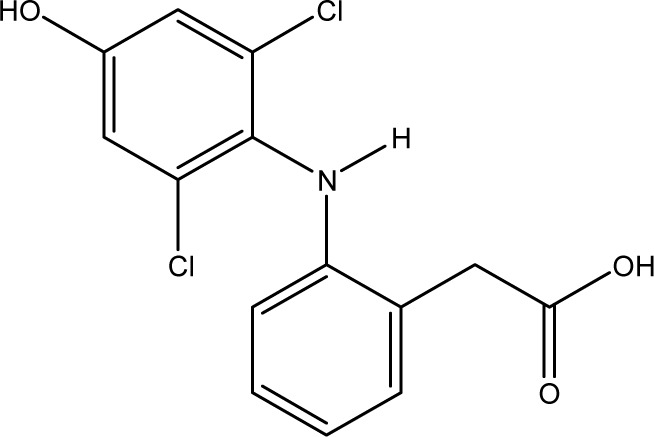
1%	
10 μg L^−1^	
4′-OH-DCF-Me	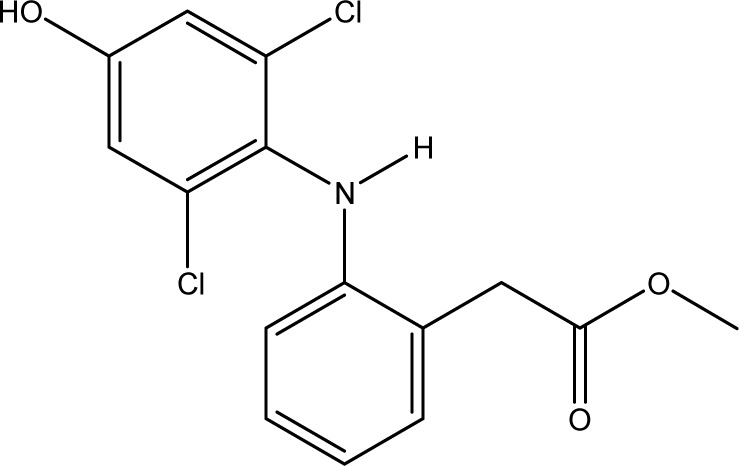
<0.01%	
100 μg L^−1^	
5-OH-DCF	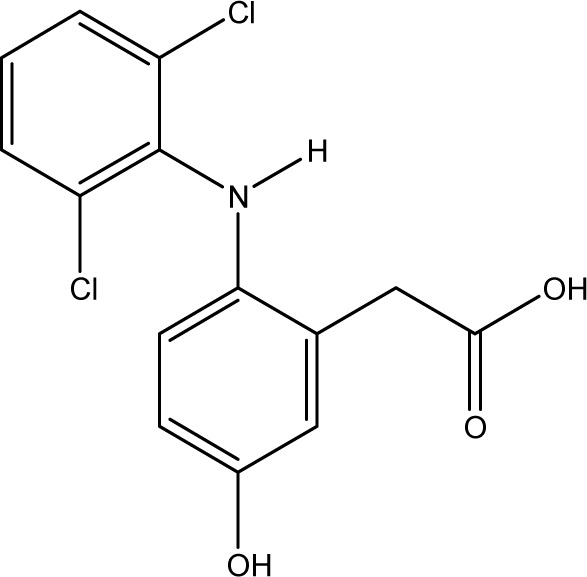
0.3%	
>100 μg L^−1^	
DCF lactam	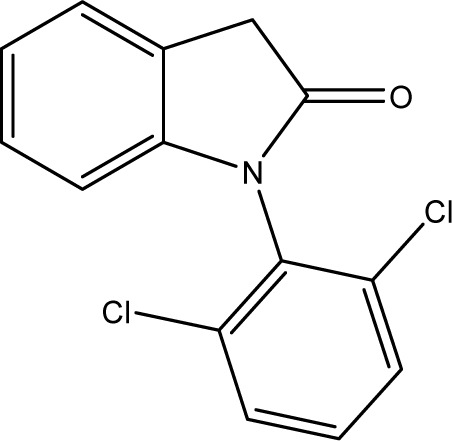
<0.01%	
1 mg L^−1^	
Tolfenamic acid	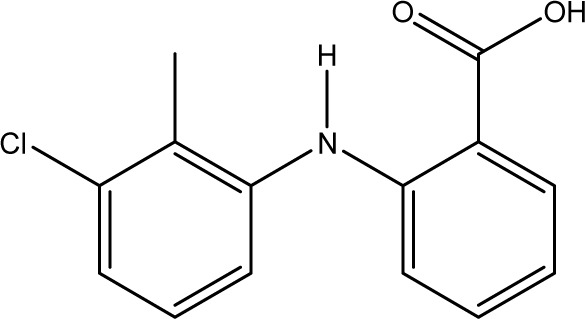
0.2%	
1 mg L^−1^	
Mefenamic acid	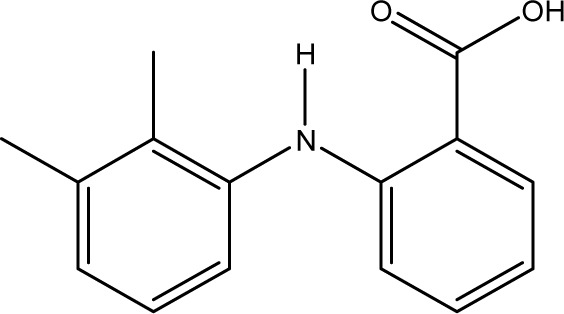
<0.01%	
1 mg L^−1^	
Photodegradation product, isolated	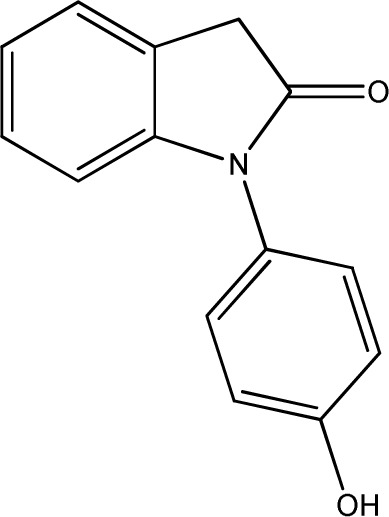
0%	
100 μg L^−1^	
Photodegradation product, isomer mixture	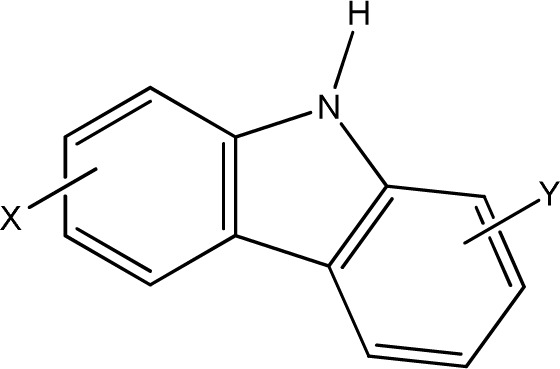
<0.01%	
1 mg L^−1^	
Photodegradation product, LC-ELISA	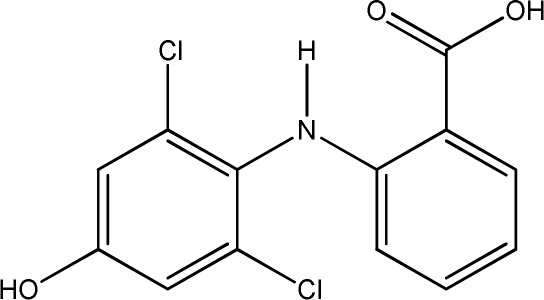
<10%	

**Fig. 12:**
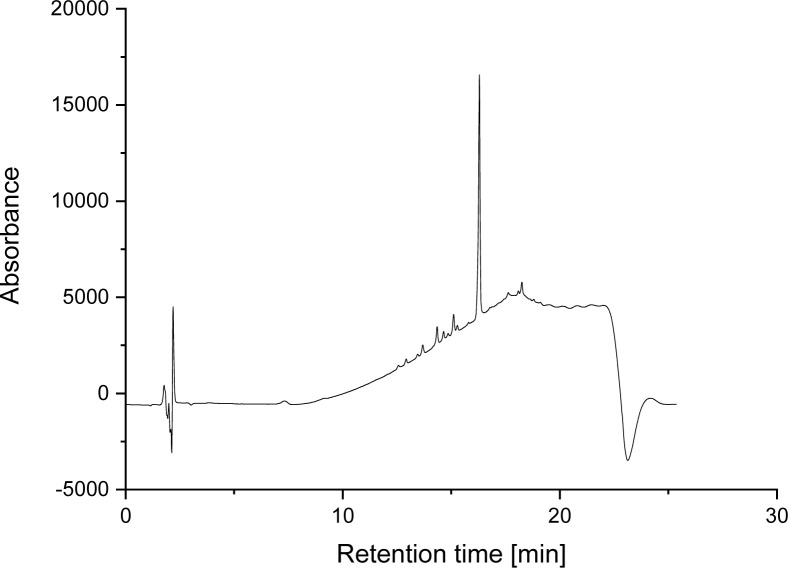
HPLC-UV chromatogram of an electrochemically generated mixture of DCF oxidation products.

LC-ELISA tests were performed on a mixture of electrochemically generated DCF oxidation products.^38^ The chromatogram of the mixture is shown in Fig. 12. The structure of the degradation products were compared with literature.^37^ Only one product resulted in a signal, which indicates a cross-reactivity, yet of less than 10%.

For mAb F01G21, the hapten DCF-Ahx has the highest cross-reactivity with 150%. Furthermore, compounds in which DCF is esterified (ACF and DCF-Me) are distinguished by high cross-reactivity (61% and 44%). The glucuronide of DCF is a natural metabolite and has a cross-reactivity of 57%, the highest value for naturally occurring DCF compounds. While DCF was previously used for the synthesis of the coating antigen, DCF-Ahx was used in this study. Since the carboxyl function has been used for protein coupling to produce an immunogen, the antibody is “blind” for this structural element. For this reason, substituents on the carboxyl function have the smallest influence on antibody binding. If, however, a substituent, for example in the form of a hydroxy function, is introduced into the aromatic ring, cross-reactivity decreases drastically (maximum 1% for 4′-OH-DCF with mAb F01G21). The decrease can be explained on the one hand with the steric change as well as the higher electron density in the aromatic system by the electron donor.

Photodegradation products show, if at all, only negligible cross-reactivity (Table 4). The same applies to pharmaceuticals that only contain structural elements of diclofenac. An overestimation by the cross-reactants requires such a high concentration that it is of no relevance in analysing environmental samples. Only the presence of 4′-OH-DCF in concentrations higher than the parent compound would markedly interfere with determination, however, in our studies in the Berlin sewage treatment plants, 4′-OH-DCF was always significantly lower in concentration, which means that no influence is to be expected.

### Application to water samples

In our HPLC-MS/MS studies (results in Table 5), DCF could be detected in waters from two lakes (Dämritzsee and Seddinsee) of the state of Brandenburg with concentrations of 3–4.4 ng L^−1^. A much higher level of 270 ng L^−1^ could be detected in Berlin's Teltowkanal at Köpenick which contains considerable amounts of wastewater. Levels of 1.9–5.2 μg L^−1^ were determined in sewage treatment plants (SWTP) of Berlin. These values correspond to those in literature.

**Table 5:** DCF concentrations determined by HPLC-MS/MS and ELISA

Sample	DCF concentration by HPLC-MS/MS	DCF concentration by ELISA	Recovery (ELISA *vs.* HPLC)
Dämritzsee (lake Dämritz)	4.4 ng L^−1^	4.0 ng L^−1^	91%
Seddinsee (lake Seddin)	3.0 ng L^−1^	3.2 ng L^−1^	107%
Teltowkanal Köpenick	270 ng L^−1^	278 ng L^−1^	103%
SWTP Waβmannsdorf influent	2.7 μg L^−1^		
SWTP Waβmannsdorf effluent	3.4 μg L^−1^	4.7 μg L^−1^	138%
SWTP Ruhleben influent	2.1 μg L^−1^		
SWTP Ruhleben effluent	1.9 μg L^−1^	2.5 μg L^−1^	133%
SWTP Münchehofe influent	3.1 μg L^−1^		
SWTP Münchehofe effluent	5.2 μg L^−1^	6.7 μg L^−1^	130%
SWTP Stahnsdorf influent	2.3 μg L^−1^		
SWTP Stahnsdorf effluent	2.9 μg L^−1^	4.1 μg L^−1^	142%

In wastewater samples, concentrations of 0.7 to 11 μg L^−1^ have been found,^31^ and in surface water samples 1 to 7 ng L^−1^,^30^ and, in a Europe-wide assessment, 0.5 to 2550 ng L^−1^.^41^ In the Danube in Hungary, DCF was found in concentrations of 59 to 442 ng L^−1^.^56^ In wastewater, in Germany, concentrations of 2.2 to 4.5 μg L^−1^ have been determined.^57^

A study found that in surface waters, diclofenac is present in concentrations of 11–310 ng L^−1^, mostly as the parent compound.^58^ In a Spanish river 57 ± 20 ng L^−1^ were found.^59^ In Italy, DCF concentrations in surface water ranged from 6.2 to 149 ng L^−1^ resulting from inputs of wastewater treatment systems.^60^ Overall, concentrations of DCF in waters worldwide ranged from 1.3 to 19,300 ng L^−1^ in surface water and, 245–2126 ng L^−1^ in wastewater treatment plant influents.^61^

In some cases, higher values were measured in the effluents than in the influents. This is because influent and effluent samples were taken almost simultaneously, therefore, the samples are not related to show elimination. ELISA studies with mAb F01G21 showed good agreement with results obtained by HPLC-MS/MS (Table 5). Surface water samples were analysed after pre-concentration by means of SPE. The pre-concentration factor for the lakes' water was 1 : 1000. The sample from the Teltowkanal had a pre-concentration factor of 1 : 100. Compared to the reference method (HPLC-MS/MS), the recovery rate was 91–104% (average 100%) for pre-concentrated surface waters. Sewage treatment plant effluents could be directly measured in ELISA and require only filtration. The results showed recoveries of 130–142% (average 136%). Overestimations can be caused by cross-reactants and matrix constituents that are not yet characterized. The results of correlations for surface water and sewage treatment plant analyses by both methods are shown in Table 6. The correlation plots are provided in the ESI (Fig. S9†). Influent samples could not be quantified by direct measurement. After pre-concentration by a factor of 100, a quantification was possible, but all samples were drastically underestimated with a recovery rate of 23–28% (data not shown). So, for influents, another sample preparation is required. One possible approach would be immunoaffinity extraction. Other studies rely on other formats, such as magnetic bead-based immunoassays.^62^ For environmental analysis, measurements of the effluents are important since they provide information on the elimination rates of the sewage treatment plants and the environmental impact *via* their input load into surface waters.

**Table 6:** Correlation of the DCF concentrations measured by HPLC-MS/MS and ELISA

Sample	Equation	*R* ^2^	Recovery
Surface waters	*y* = 1.02*x* + 0.17	0.9983	100% ± 7%
Sewage treatment plant effluents	*y* = 1.25*x* + 0.31	0.9907	136% ± 6%

## Conclusions

A series of monoclonal antibodies against diclofenac (DCF) was produced by coupling the analyte without a spacer to a carrier protein. In all assessed systems, sensitivity could be increased by introducing a spacer between analyte and the carrier protein of the coating antigen. Here, anti-DCF mAb F01G21, together with the coating antigen ACF-APO, were for the first time employed for an indirect competitive ELISA for environmental analysis. With this assay, samples of sewage treatment plant effluents can be measured directly after filtration. The results agreed well with HPLC-MS/MS analyses. Surface and drinking water samples can, thus, be measured after pre-concentration by means of solid-phase extraction. The antibody mAb F01G21 is very robust against matrix influences such as organic solvents, salt content, NOM and pH value. The metabolite DCF-acyl glucuronide was identified as a relevant cross-reactant. For the first time, cross-reactivity investigations were carried out on DCF photodegradation products. Among the photodegradation products one cross-reactant was isolated and identified. With a threshold value of 100 μg L^−1^ it will not affect real-world sample determination.

## Reagent availability

The anti-DCF antibody mAb F01G21 has been licensed to sifin diagnostics gmbh (http://www.sifin.de/) from where it can be obtained.

## Conflicts of interest

The authors declare that they have no conflicts of interest to disclose.

## Supplementary Material

AY-016-D3AY01333B-s001
