# Supplementary material for: Re-assessment of monoclonal antibodies against diclofenac for their application in the analysis of environmental waters
Source: Anal Methods. 2024 May 14;16(21):3349–63. doi: 10.1039/d3ay01333b (PMC11138808; doi:10.1039/d3ay01333b)
Supplement: AY-016-D3AY01333B-s001 [file AY-016-D3AY01333B-s001.pdf]

Electronic Supplementary Material (ESI) for Analytical Methods.
This journal is © The Royal Society of Chemistry 2024

Supplementary Information

Re-assessment of monoclonal antibodies against diclofenac for their application in the analysis of environmental waters

Stephan Schmidt,[a,b] Holger Hoffmann,[a,c] Leif-Alexander Garbe,[d] Andrea Harrer,[e,f,g] Markus Steiner,[e,h] Martin Himly[e] and Rudolf J. Schneider[a,b]

a) BAM Federal Institute for Materials Research and Testing, Department of Analytical Chemistry; Reference Materials, Richard-Willstätter-Str. 11, 12489 Berlin, Germany. E-mail: rudolf.schneider@bam.de; Tel: +49 3081041151

b) Technische Universität Berlin, Straße des 17. Juni 135, D-10623 Berlin

c) Humboldt-Universität zu Berlin, Department of Chemistry, Brook-Taylor-Str. 2, D-12489 Berlin, Germany

d) Hochschule Neubrandenburg, Fachbereich Agrarwirtschaft und Lebensmittelwissenschaften, D-17033 Neubrandenburg, Germany

e) Paris Lodron University of Salzburg, Department of Biosciences and Medical Biology, Division of Allergy and Immunology, A-5020 Salzburg, Austria

f) Current address: Paracelsus Medical University, Department of Neurology, A-5020 Salzburg, Austria

g) Current address: Paracelsus Medical University, Department of Dermatology and Allergology, A-5020 Salzburg, Austria

h) Current address: Salzburg Cancer Research Institute, Laboratory for Immunological and Molecular Cancer Research (SCRI-LIMCR), A-5020 Salzburg, Austria

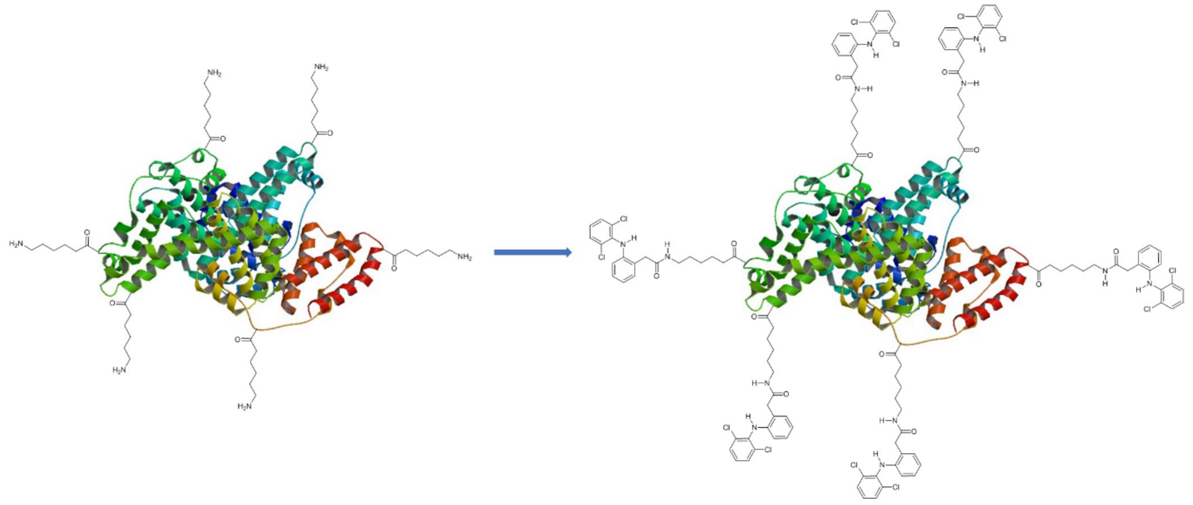

Fig. S1 Direct coupling of DCF to 6-Ahx-BSA, avoiding the need for producing a spacer derivative of DCF beforehand

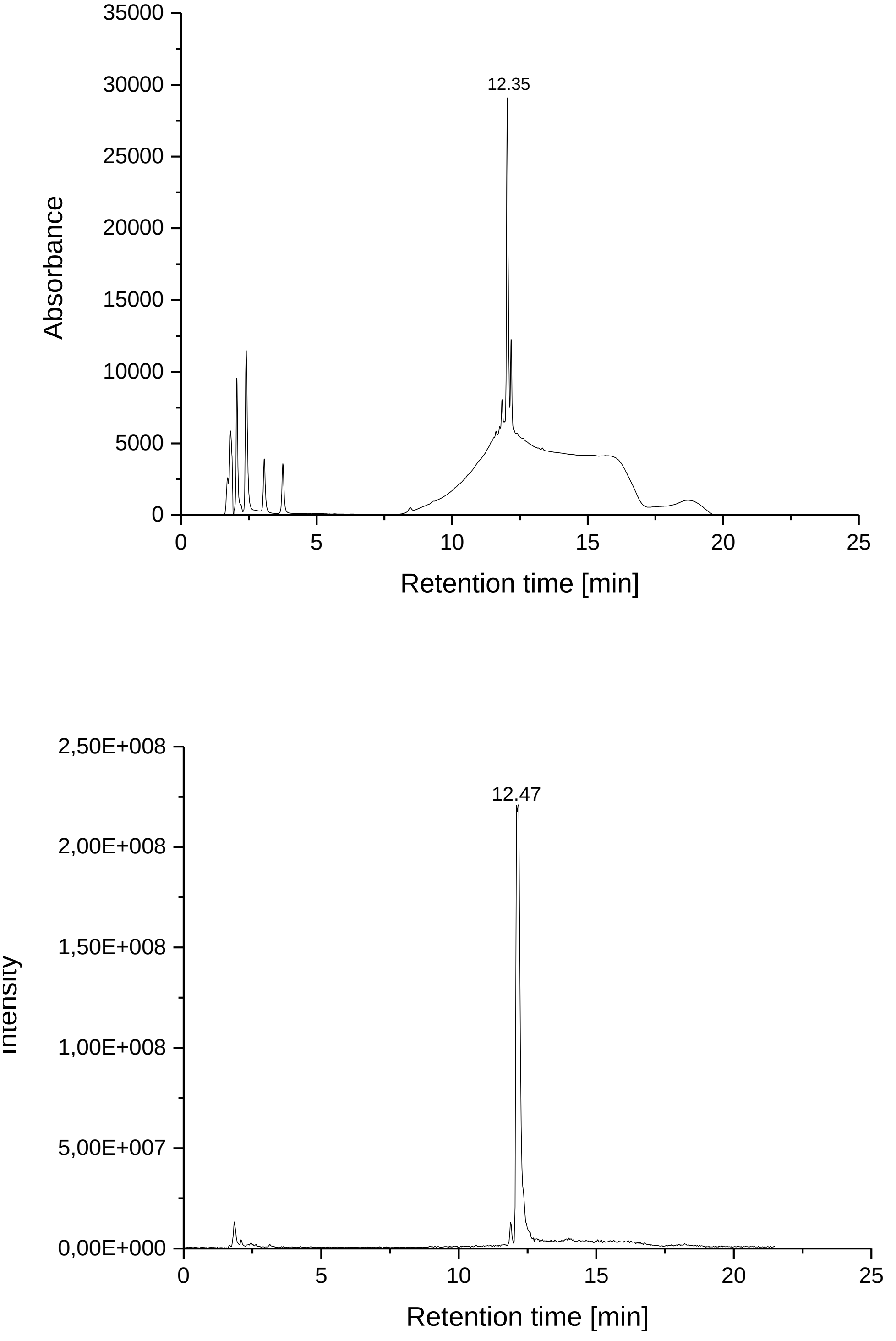

Fig. S2 HPLC chromatograms of the enzymatic digest of DCF-6-Ahx-BSA (by protease from *Streptomyces griseus*), showing in the UV/Vis trace (upper panel), and *m/z*=537 SIM trace (lower panel) the signals for DCF-6-Ahx-Lys at 12.35/12.47 min.

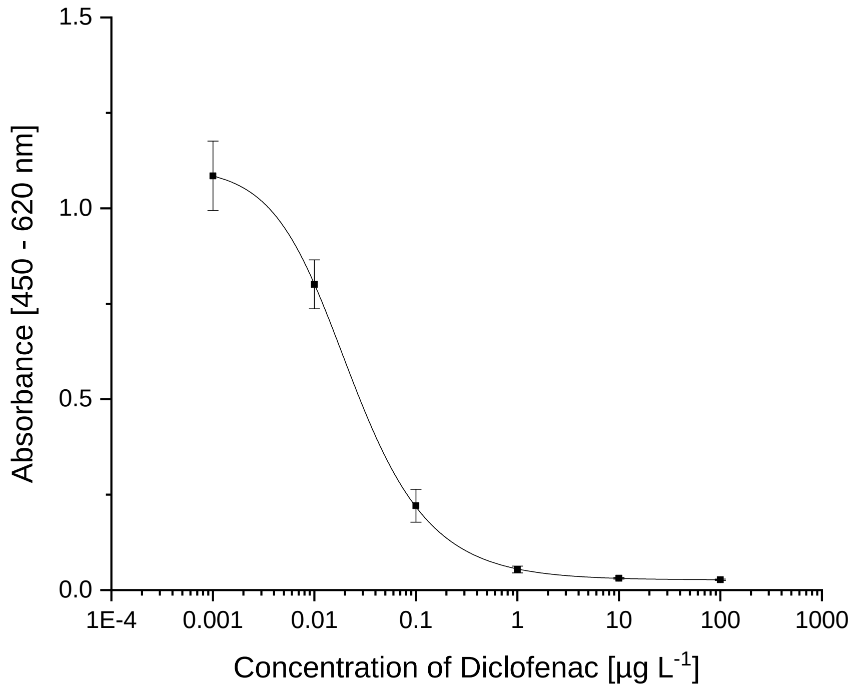

Fig. S3 Calibration curve of an indirect ELISA for DCF after coating with the synthesized DCF-6-Ahx-BSA (1:40 000). Signal is created via a sandwich of anti-DCF mAb 12G5 [10] (1:32 000) and an HRP-labelled anti-mouse secondary Ab (secAb1-HRP, 1:40 000)

A

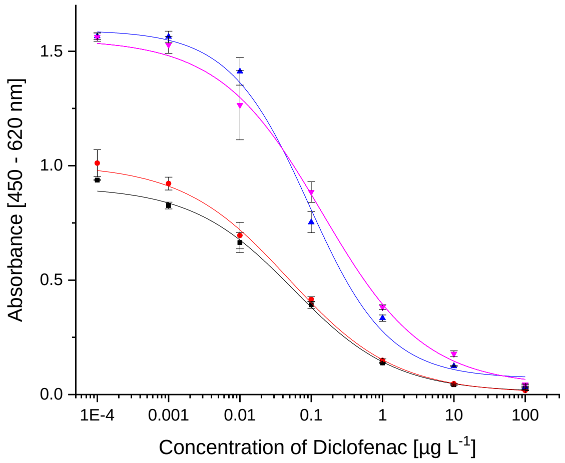

B

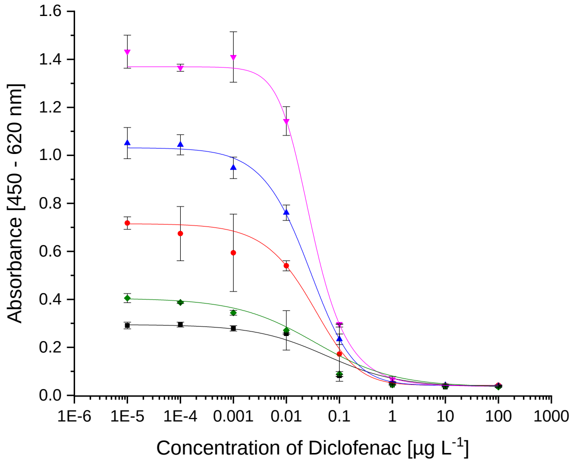

C

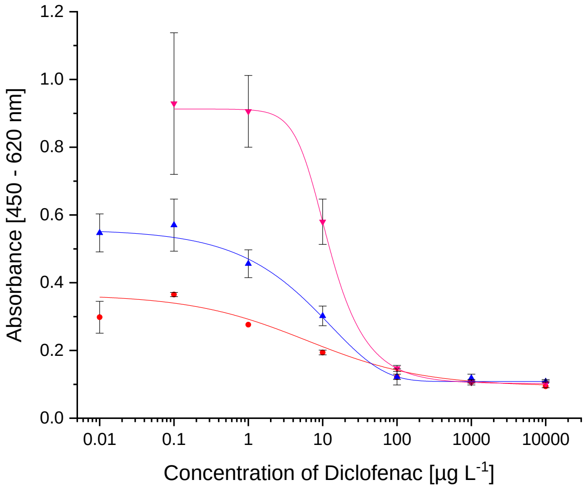

Fig. S4 Calibration curves for DCF with different coating antigens (■DCF-OVA, ●DCF-6-Ahx-OVA, ▲DCF-6-Ahx-APO, ▼ACF-APO, ♦DCF-6-Ahx-BSA, diluted 1:40 000 (A and C) and 1:50 000 (B) in PBS), different primary antibodies (A: pAb1 1:32 000; B: mAb 12G5 1:10 000; C: mAb F01G21 1:10 000; all dilutions in TRIS) and different secondary antibodies (A: secAb2-HRP 1:40 000; B: secAb1-HRP 1:20 000; C: secAb1-HRP 1:40 000; all dilutions in PBS). All three primary antibodies were assessed with all five coating antigens. In cases, where no curve was obtained, it was omitted from the plots.

Table S1

Elemental composition of the NOM (natural organic matter) employed [53]

Element	Große Fuchskuhle	Schwarzer See
C [mg g^{-1}]	343.7	210.4
H [mg g^{-1}]	38.5	25.7
N [mg g^{-1}]	11.2	5.8
S [mg g^{-1}]	27.9	45.8
Fe [mg g^{-1}]	2.53	0.12
Mn [mg g^{-1}]	0.15	0.09
Zn [mg g^{-1}]	0.06	0.02
Cu [mg g^{-1}]	0.06	0.04
Al [mg g^{-1}]	1.75	0.05
K [mg g^{-1}]	7.8	8.8
Na [mg g^{-1}]	93	175
Ca [mg g^{-1}]	6.4	24.9
Ash content [%]	43.3	74.4
Water content [%]	7.3	6.0

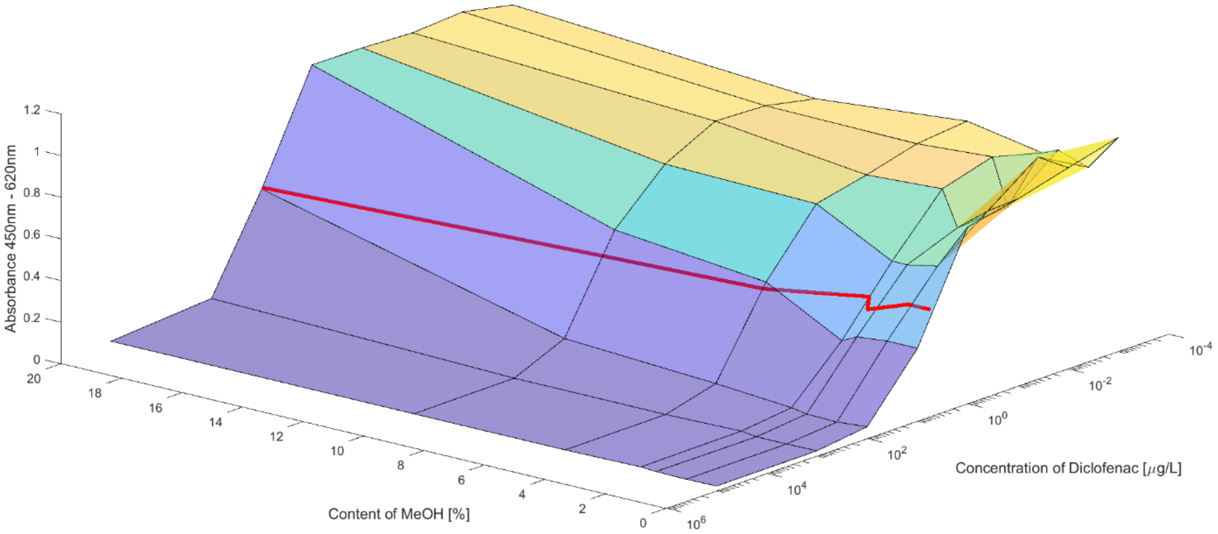
Absorbance 450nm - 620nm
Content of MeOH [%]
Concentration of Diclofenac [μg/L]

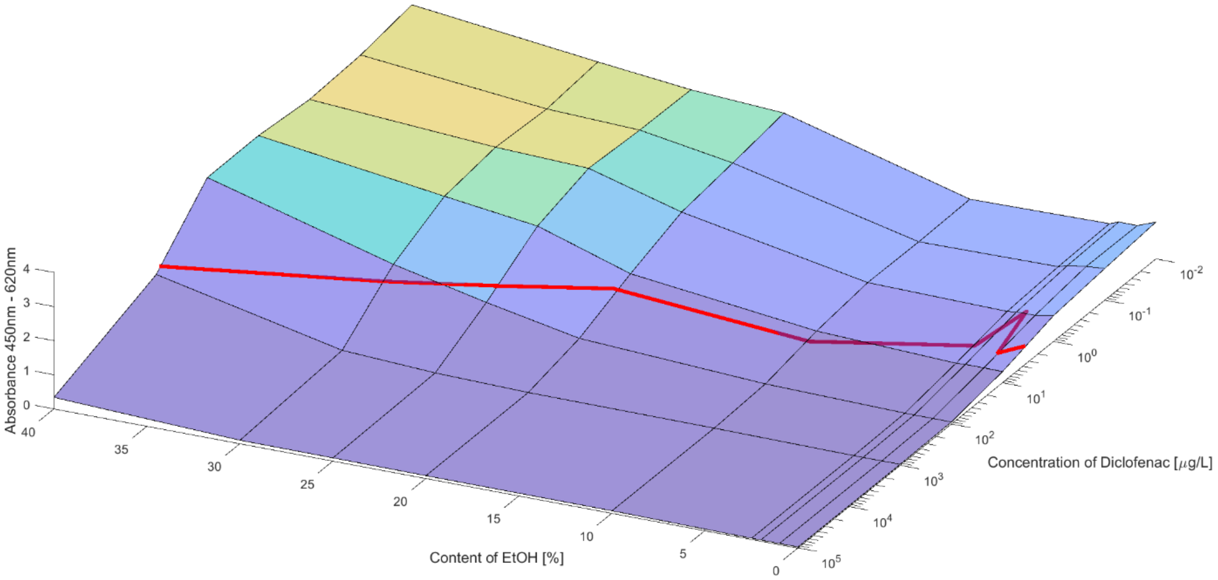
Absorbance 450nm - 620nm
Content of EtOH [%]
Concentration of Diclofenac [μg/L]

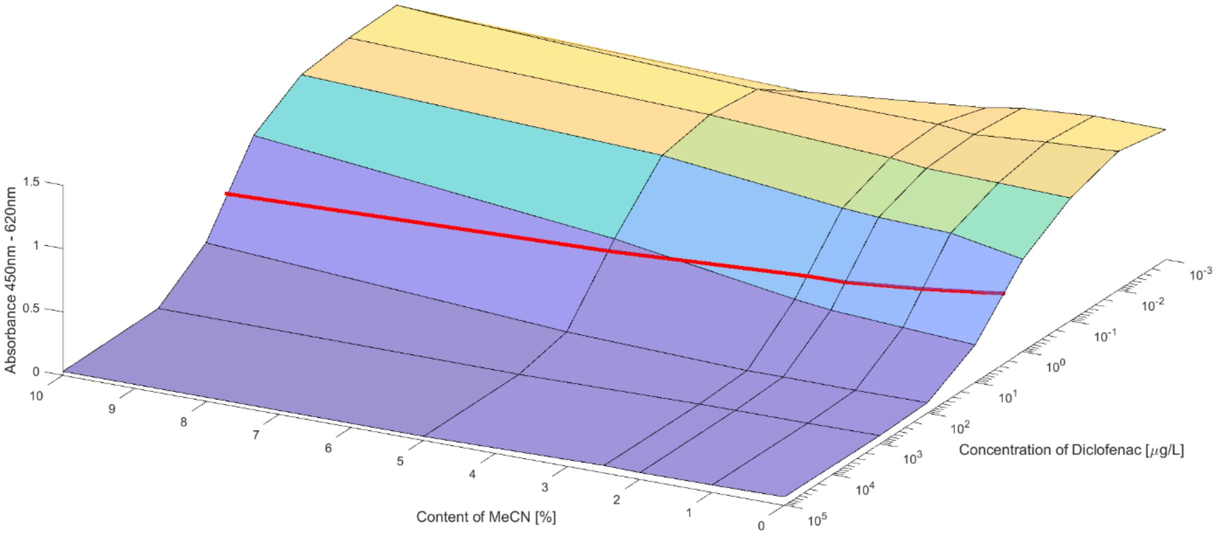
Absorbance 450nm - 620nm
Content of MeCN [%]
Concentration of Diclofenac [μg/L]

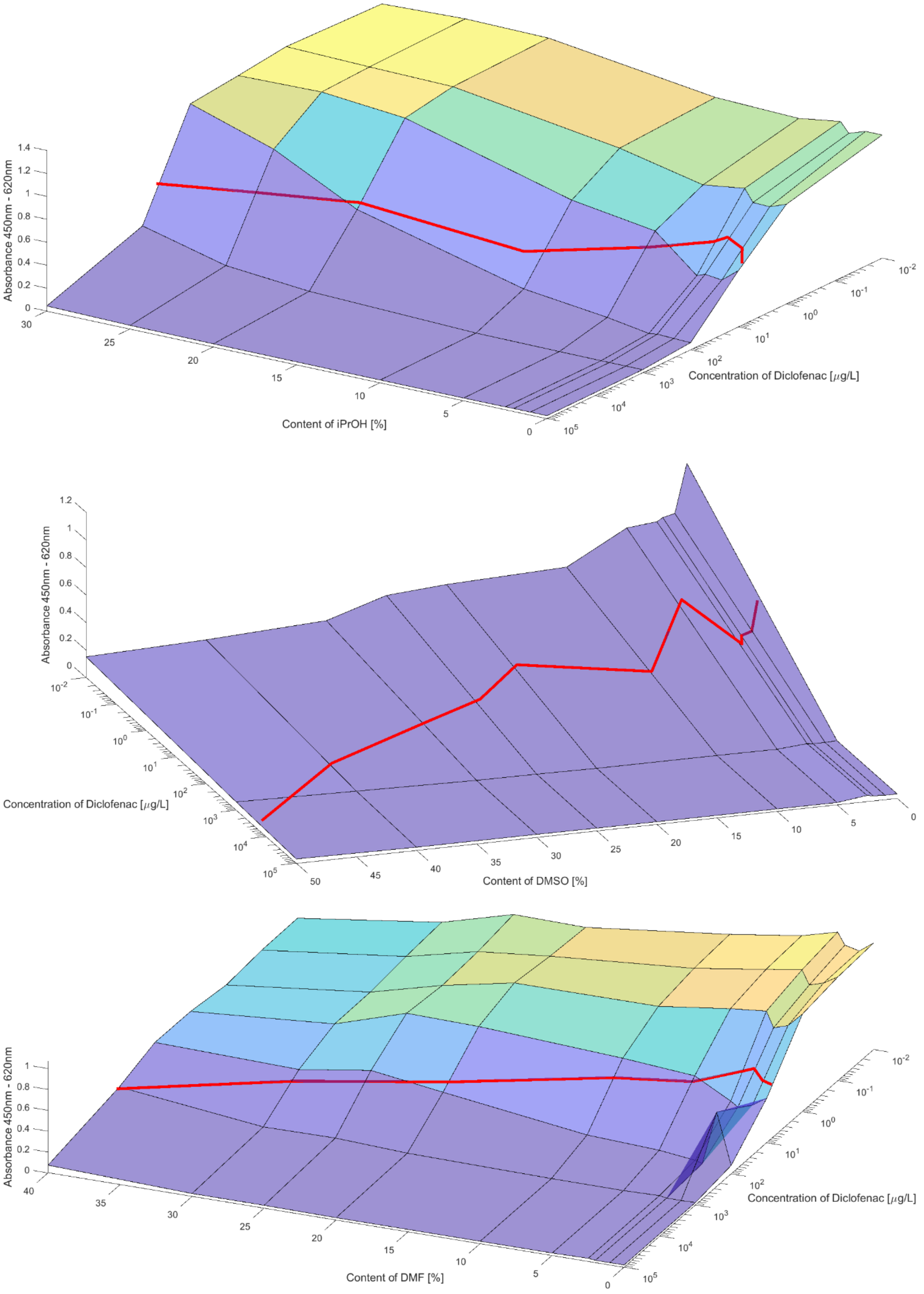

Fig. S5 ELISA calibration functions for DCF with increasing solvent share in the calibrators in a 3D plot (from top to bottom: methanol, ethanol, acetonitrile, isopropanol, DMSO, DMF). The red line connects test midpoints (IC_{50} values) and helps to illustrate trends in the sensitivity of the assays.

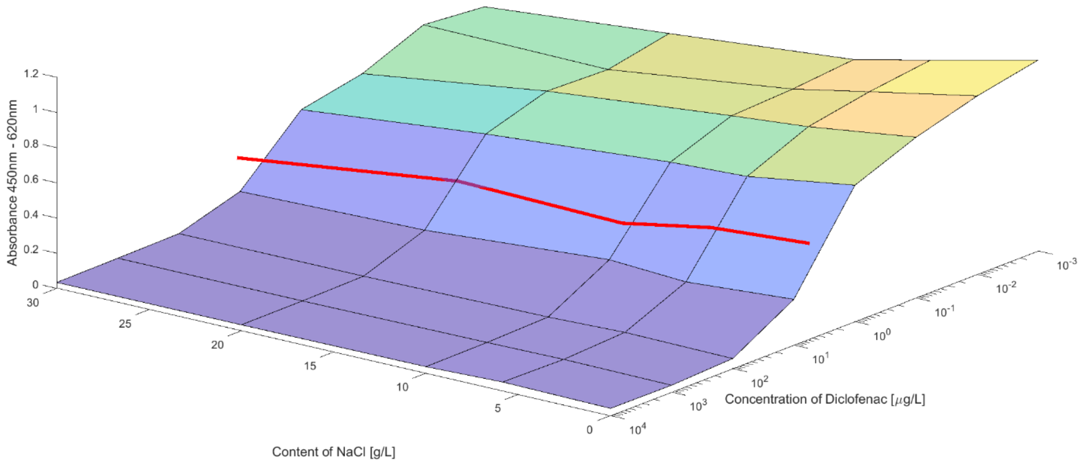

Fig. S6 ELISA calibration function for DCF with increasing concentration of sodium chloride in the calibrators in a 3D plot. The red line connects test midpoints, indicating little influence of salt concentration on this parameter, i.e., on assay sensitivity.

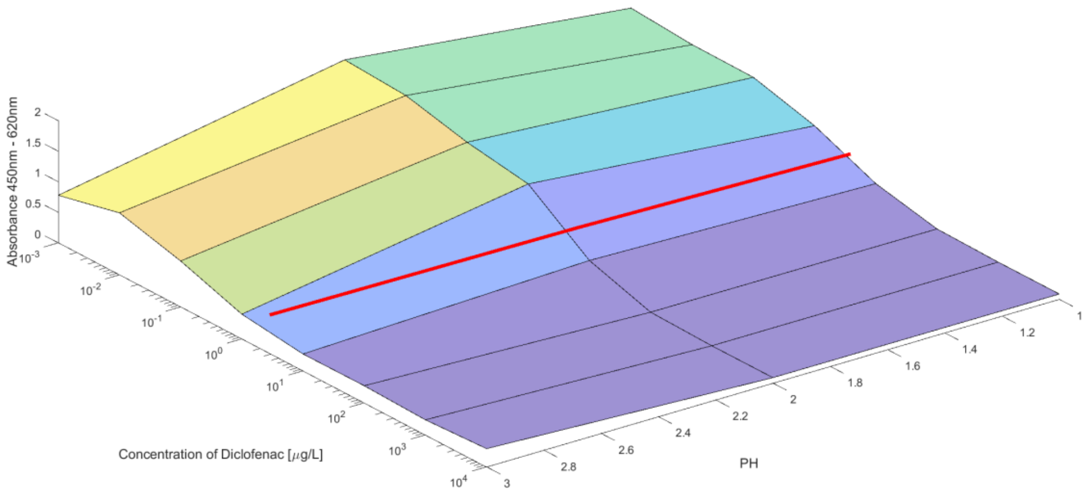

Fig. S7 pH dependency of the DCF calibration function in the acidic range in a 3D plot. The red line connects test midpoints, indicating little dependence on sample pH.

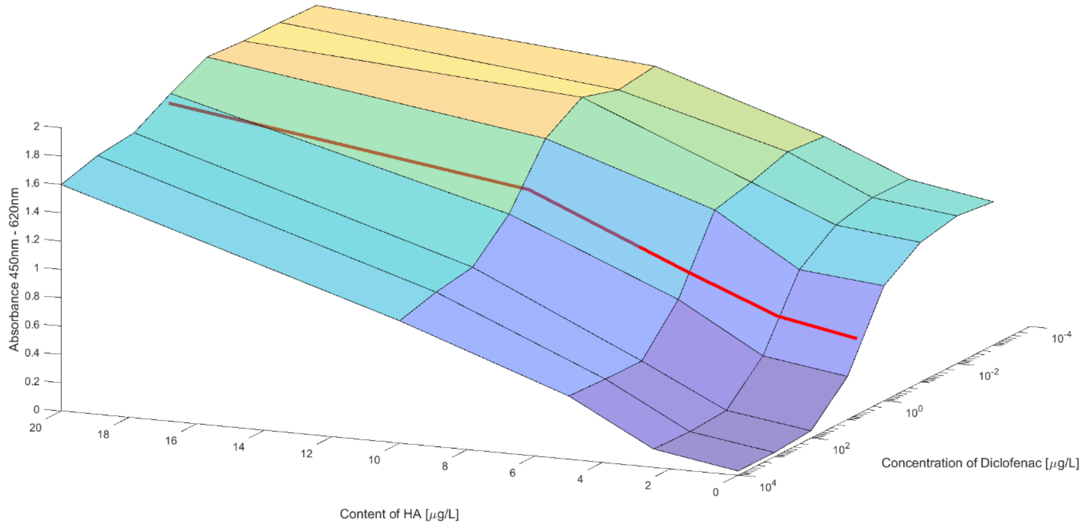

Fig. S8 ELISA calibration function for DCF with increasing humic acid (HA) content in a 3D plot. The red line connects test midpoints and illustrates the severe changes starting right above 2.5 mg/L humic acid, meaning a strong influence of humic acid content in samples.

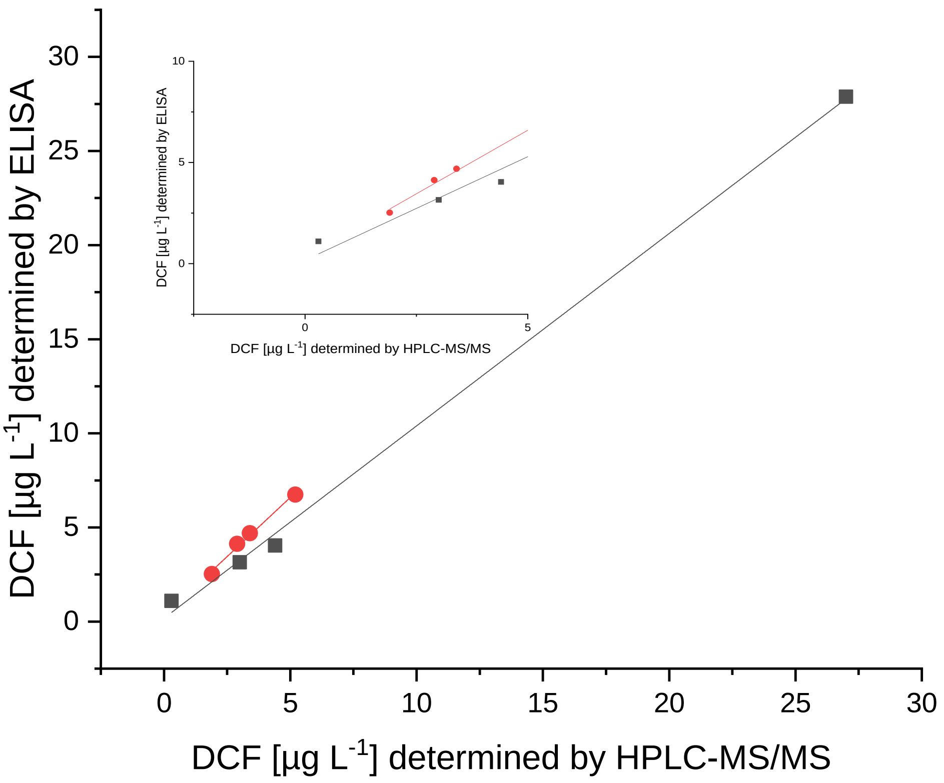

Fig S9 Correlation between ELISA and HPLC-MS/MS results (■ Surface water, ● Wastewater)

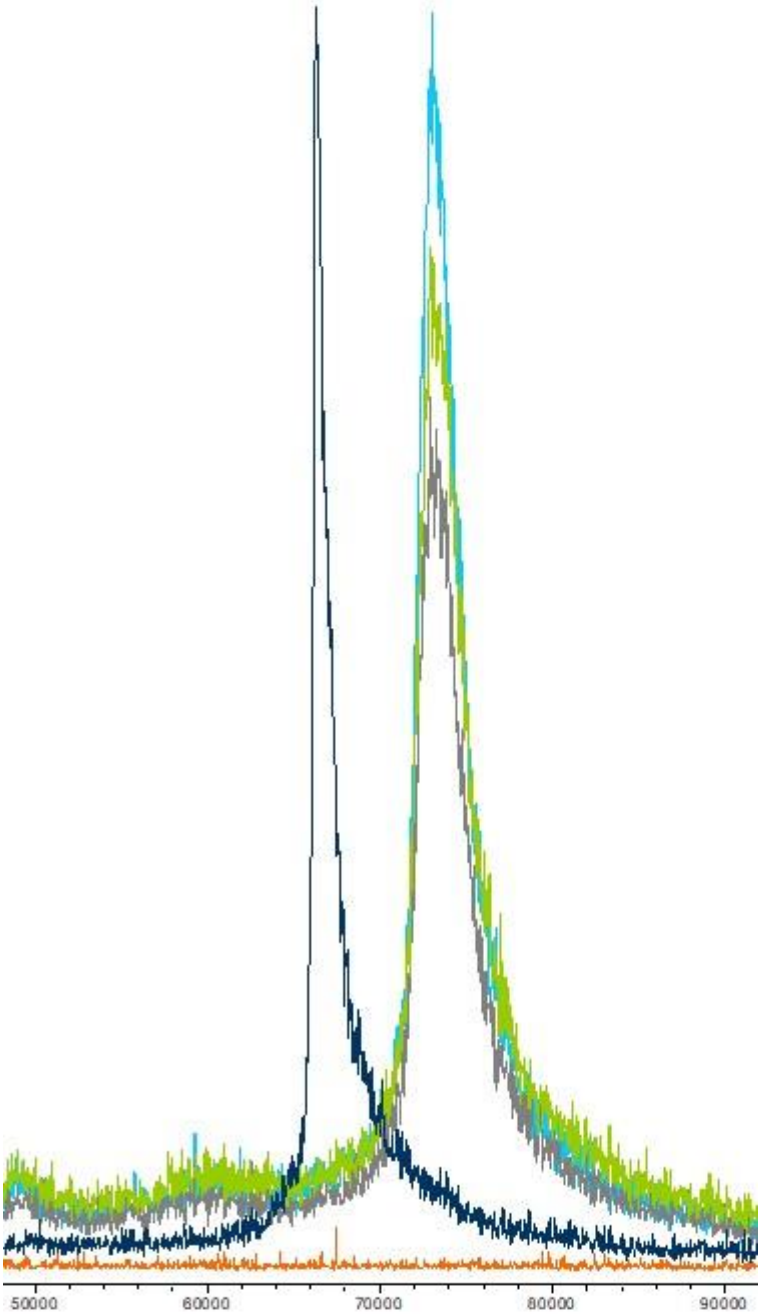

Fig S10 MALDI-ToF-MS spectra (detail) of Boc-6-Ahx-BSA (average mass peak at m/z 76,668 Da, green), deprotected Boc-6-Ahx-BSA (= 6-Ahx-BSA, 71,280 Da, grey), and DCF-6-Ahx-BSA (75,254 Da, light blue). Average mass peak of BSA at m/z 66,411 Da (dark blue).

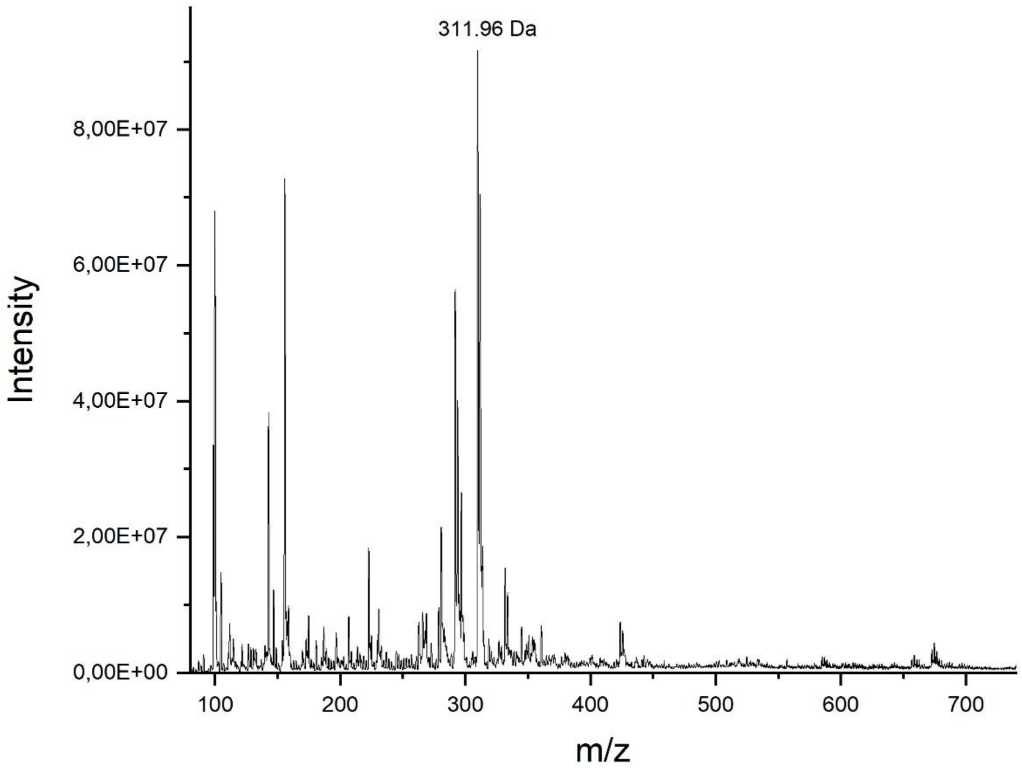

Fig. S11 MS spectrum of DCF-Me (base peak at m/z = 311.96 Da confirming the identity of the compound).

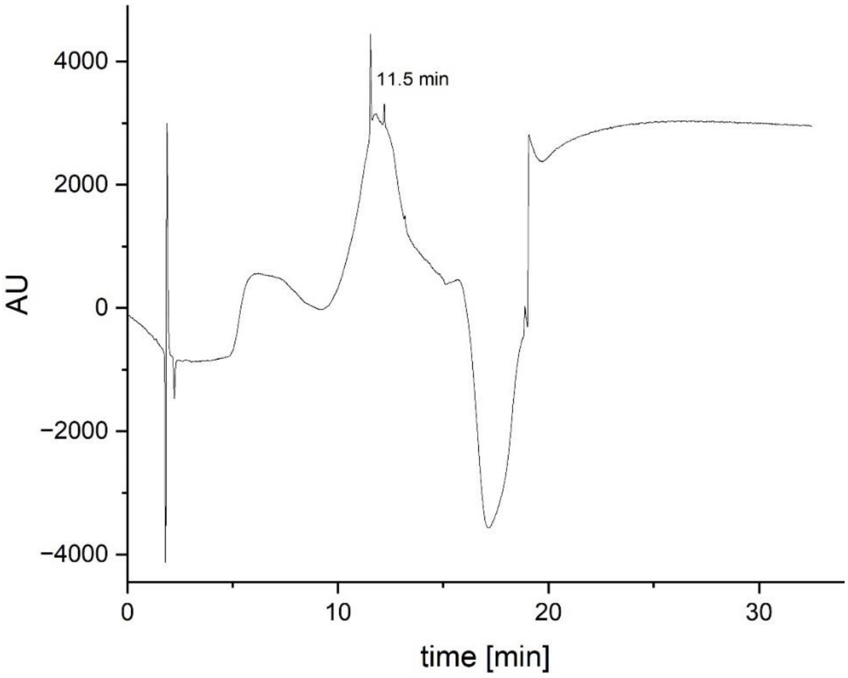

Fig. S12 UV trace of the LC separation of the photodegradation product. The product elutes around 11.5 min.

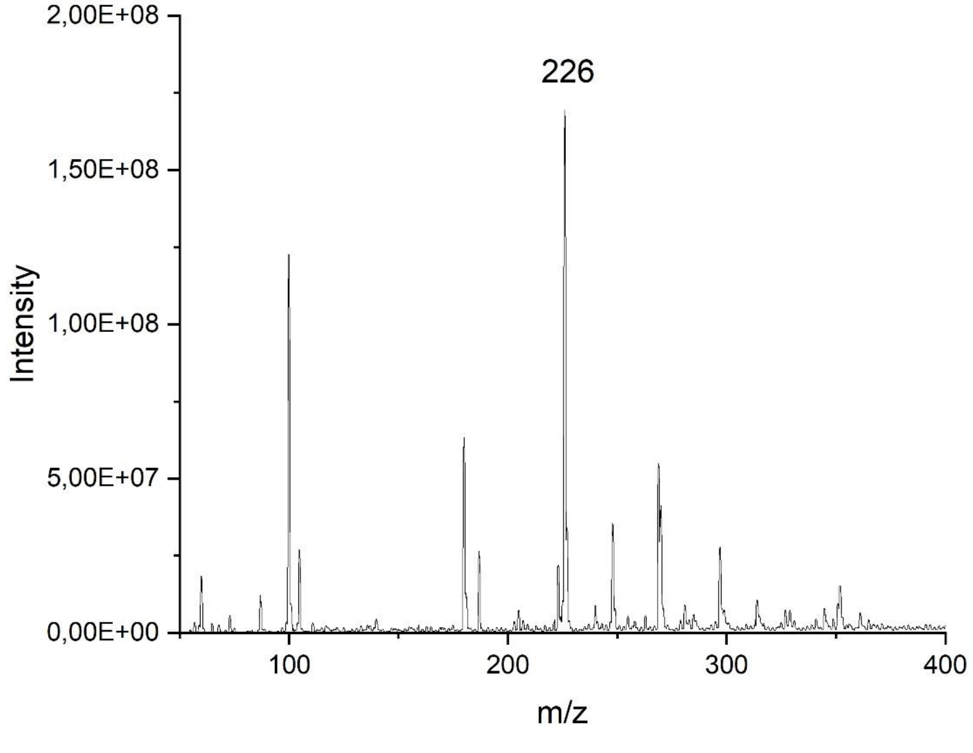

Fig. S13 Mass spectrum of isolated photodegradation product ($C_{14}O_2NH_{11}$). Identity is confirmed by its base peak at m/z = 226 (M+H).
